# A robust pipeline for efficient knock-in of point mutations and epitope tags in zebrafish using fluorescent PCR based screening

**DOI:** 10.1186/s12864-022-08971-1

**Published:** 2022-12-07

**Authors:** Blake Carrington, Ramanagouda Ramanagoudr-Bhojappa, Erica Bresciani, Tae-Un Han, Raman Sood

**Affiliations:** 1 Translational and Functional Genomics Branch, Zebrafish Core, National Human Genome Research Institute, National Institutes of Health, Bethesda, MD 20892 USA; 2 Cancer Genetics Unit, Cancer Genetics and Comparative Genomics Branch, National Human Genome Research Institute, National Institutes of Health, Bethesda, MD 20892 USA; 3 Oncogenesis and Development Section, Translational and Functional Genomics Branch, National Human Genome Research Institute, National Institutes of Health, Bethesda, MD 20892 USA; 4 Molecular Neurogenetics Section, Medical Genetics Branch, National Human Genome Research Institute, National Institutes of Health, Bethesda, MD 20892 USA

**Keywords:** Zebrafish, CRISPR/Cas9, Knock-in, Epitope tags, Point mutations, ssODN

## Abstract

**Background:**

Genome editing using CRISPR/Cas9 has become a powerful tool in zebrafish to generate targeted gene knockouts models. However, its use for targeted knock-in remains challenging due to inefficient homology directed repair (HDR) pathway in zebrafish, highlighting the need for efficient and cost-effective screening methods.

**Results:**

Here, we present our fluorescent PCR and capillary electrophoresis based screening approach for knock-in using a single-stranded oligodeoxynucleotide donor (ssODN) as a repair template for the targeted insertion of epitope tags, or single nucleotide changes to recapitulate pathogenic human alleles. For the insertion of epitope tags, we took advantage of the expected change in size of the PCR product. For point mutations, we combined fluorescent PCR with restriction fragment length polymorphism (RFLP) analysis to distinguish the fish with the knock-in allele. As a proof-of-principle, we present our data on the generation of fish lines with insertion of a FLAG tag at the *tcnba* locus, an HA tag at the *gata2b* locus, and a point mutation observed in Gaucher disease patients in the *gba* gene. Despite the low number of germline transmitting founders (1–5%), combining our screening methods with prioritization of founder fish by fin biopsies allowed us to establish stable knock-in lines by screening 12 or less fish per gene.

**Conclusions:**

We have established a robust pipeline for the generation of zebrafish models with precise integration of small DNA sequences and point mutations at the desired sites in the genome. Our screening method is very efficient and easy to implement as it is PCR-based and only requires access to a capillary sequencer.

**Supplementary Information:**

The online version contains supplementary material available at 10.1186/s12864-022-08971-1.

## Background

Zebrafish are a popular vertebrate model system for functional genomics and human disease modelling studies due to their external fertilization, optically transparent embryos, high fecundity, rapid embryonic development, evolutionarily conserved biological pathways, and availability of methods for genetic manipulations [1–3]. Recent advances in targeted genome editing technologies, especially Clustered Regularly Interspaced Short Palindromic Repeats (CRISPR)/Cas9, have made it relatively easy to generate desirable gene knockout models in zebrafish [4–8]. These methods take advantage of the error-prone non-homologous end joining (NHEJ) pathway that is activated by the double stranded break (DSB) caused by the CRISPR/Cas9 [9]. Our lab and others have developed high-throughput and cost-effective protocols to generate single or multiple gene knockouts in zebrafish using CRISPR/Cas9 [5, 7, 10–14]. Similarly, CRISPR/Cas9 mediated targeted mutagenesis has been used to generate fish models with targeted knock-in of desired exogeneous sequences by providing a DNA repair template to activate homology-directed repair (HDR) pathway [15]. A variety of repair templates, such as, single-stranded oligodeoxynucleotides (ssODNs) [4, 11, 16–25], long single or double stranded DNA [26–28], or plasmid DNA [29–31] have been shown to work in zebrafish and their choice depends on the size of the cargo to be inserted. Examples of fish lines generated using targeted knock-in include addition of fluorescent reporters for real time analysis of gene expression [26, 29–31], epitope tags for protein level analyses [19, 23, 27], loxP sites for conditional gene knockouts [18, 20] and nucleotide substitutions for analysis of disease-specific point mutations [16, 21, 22, 24].

Targeted knock-in by HDR still remains a challenging process in zebrafish as HDR is highly inefficient compared to NHEJ, therefore, extensive screening is required to identify the rare founder fish that transmit a precisely integrated repair template to their progeny [16, 22]. Furthermore, knock-in events are often imprecise either due to the presence of simultaneous indels caused by NHEJ or due to errors during recombination [4, 17, 19, 20, 23]. Screening for precise knock-in is especially difficult when using ssODNs due to the lack of visual tools, like fluorescent reporters, which can be used for knock-in with larger repair templates [26, 30, 31]. Therefore, expensive and labor-intensive approaches, such as cloning and sequencing of a large number of clones or next-generation sequencing (NGS) of pooled embryos are used to determine the success of knock-in using ssODNs [4, 11, 17, 21, 23, 24]. NGS requires access to special equipment and bioinformatic expertise for processing the large amounts of sequencing data, thus posing a challenge for many laboratories. While online tools, such as, TIDER (Tracking of Insertions, Deletions and Recombination events) and ICE (Inference of CRISPR Edits) can be used to determine the knock-in efficiency and infer the edited sequences from Sanger sequencing data, these methods require high quality sequence reads. If a particular nucleotide is not represented well in the chromatogram it can lead to errors in the inferred sequence, especially with knock-in for point mutations [32, 33]. Recently, Prykhozhij and colleagues [24] showed that a combination of allele-specific PCR (AS-PCR) with restriction digest accurately identifies true knock-in from off-target trans knock-in events for point mutations. However, AS-PCR is a gel-based method and thus difficult to scale up.

Thus, the goal of our study was to develop robust screening methods for knock-in using ssODNs that can be easily implemented by others. Previously, we had developed capillary electrophoresis-based fragment separation of fluorescent PCR products for accurate genotyping of knockout fish lines with indels and its modification, termed CRISPR-STAT (CRISPR Somatic Tissue Activity Test) for the evaluation of sgRNA activity [6, 34]. Here, we describe how we adapted fluorescent PCR and CRISPR-STAT for somatic and germline screening to detect precise integration of ssODNs. To demonstrate our pipeline, we have generated stable fish lines with a FLAG tag at the 3’ end of *tcnba*, an HA tag at the 3’ end of *gata2b* and a Gaucher disease patient-specific point mutation in the *gba* gene. For insertion of epitope tags, we took advantage of knowing the exact change in size of the PCR product expected by the insertion of the repair template and used CRISPR-STAT to detect the expected size peaks among the CRISPR/Cas9-induced indels. Since no change in the size of PCR product is expected when ssODNs are used for knock-in of point mutations, we combined CRISPR-STAT with a restriction enzyme digest followed by fluorescent RFLP analysis to identify the knock-in events. Our methods make valuable additions to the genome editing toolbox for zebrafish researchers interested in generating model systems for functional genomics and disease modelling of human genetic disorders.

## Results

### Experimental design for knock-in of epitope tags

Commercial antibodies against zebrafish proteins are not readily available and cross reactivity with antibodies from other species can be hit or miss [35]. Therefore, doing any protein level analysis in zebrafish requires considerable effort to identify appropriate antibodies without any guarantee of success. To overcome these limitations, the preferred approach is to insert an epitope tag at the 5’ or 3’ end of genes by targeted knock-in using ssODNs for HDR [23, 27, 36]. Here, we demonstrate our fluorescent PCR and CRISPR-STAT based screening approach for knock-in of epitope tags by two examples: insertion of a FLAG tag at the *tcnba* locus and insertion of an HA tag at the *gata2b* locus. In both examples, we chose to insert epitope tags at the 3’ ends of the coding sequence to avoid disrupting gene function in injected fish due to CRISPR induced indels at 5’ ends.

### Knock-in of FLAG tag at 3’ end of *tcnba*

**Fig. 1:**
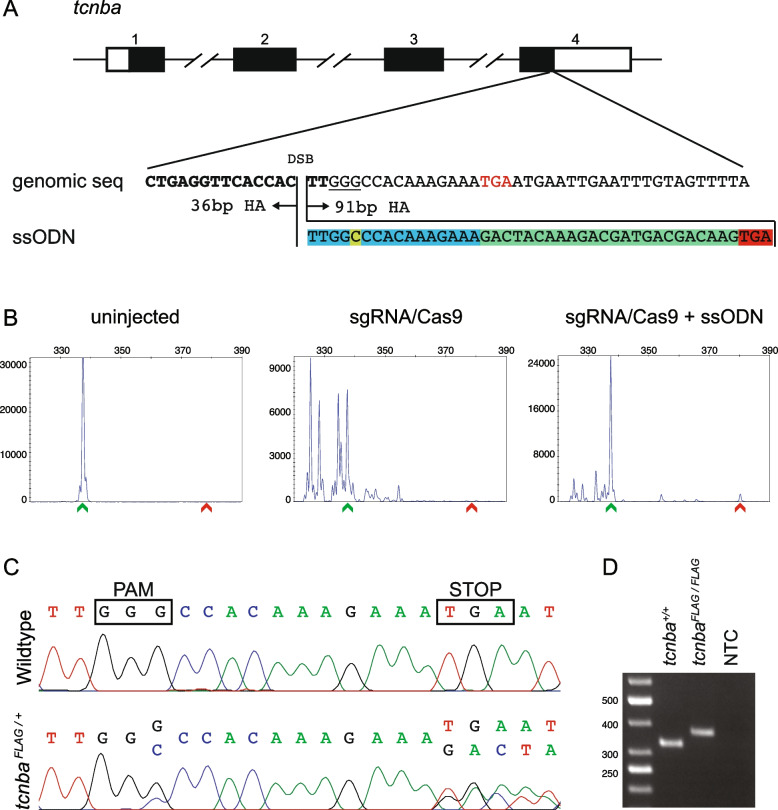
Design, screening, and validation of knock-in of FLAG tag at 3’ end of *tcnba*. **A** Schematic of *tcnba* genomic structure with coding region shaded in black (top panel), alignment of genomic sequence and ssODN template with direction of 36 bp and 91 bp homology arms (HA) marked. The ssODN contains a spacer (highlighted in blue) to maintain the ORF of *tcnba* as the double stranded break occurs 16 bp upstream of the stop codon (TGA, marked in red), a modification to the PAM site (G > C, highlighted in yellow) to prevent recutting after integration and the FLAG tag to be inserted (highlighted in green). **B** Representative CRISPR-STAT plots of uninjected, sgRNA/Cas9, and sgRNA/Cas9 plus ssODN injected embryos with X-axis showing the size of the peaks, Y-axis showing the peak height, and the size of the expected WT allele denoted by a green arrowhead. These plots were quantified for the presence of a peak generated by the insertion of ssODN (denoted by red arrowhead). **C** Sequence chromatograms showing WT and a positive F1 embryo to confirm germline transmission of knock-in sequence. **D** An agarose gel image showing expression of the FLAG tag at *tcnba* locus by RT-PCR. RNA from *tcnba*^+*/*+^ or *tcnba*^*FLAG/FLAG*^ embryos were used as template and water was used as the no template control (NTC). The original gel image is provided in additional file 2: Fig. 1 – Supporting data and its cropped version is shown here

Tcnba was recently identified as one of the three cobalamin transport proteins (Tcn2, Tcnba, Tcnbb), which function in a tissue-specific manner in the zebrafish [37]. To elucidate its specific role in the cobalamin binding and transport, we sought to mark the *tcnba* locus by a FLAG tag. Therefore, we first evaluated the activity of two sgRNAs near the stop codon of *tcnba* by CRISPR-STAT and selected the highly active sgRNA-T2 for the knock-in experiments (Additional file 1: Figure S1A-B). Next, we designed the ssODN for repair template based on sgRNA-T2 as follows: 1) To maintain the entire coding region of *tcnb*a and open reading frame (ORF) for the expression of FLAG tag, we added the 16 nucleotides from the Cas9 cut site to the stop codon into the ssODN followed by the FLAG tag sequence (Fig. 1A, Additional file 1: Figure S2A). 2) We introduced a modification of the PAM site (G > C) as a CRISPR/Cas-blocking mutation (Additional file 1: Figure S2A). Following injections, CRISPR-STAT analysis was performed on uninjected, sgRNA/Cas9, and sgRNA/Cas9 plus ssODN injected embryos collected at 1 day post fertilization (dpf) (Fig. 1B) and plots were analyzed for the presence of the peak corresponding to the expected size of the knock-in allele (wildtype (WT) PCR product size + 43 bp due to integration of the ssODN). Since only the size of the PCR product is being assessed in this assay, not the nucleotide sequence, sgRNA/Cas9-injected embryos were used as control to estimate the likelihood of indels from NHEJ that would lead to a similar size product. We observed a peak at the expected size in 1 out of 26 embryos injected with sgRNA/Cas9 alone in comparison to 6 out of 40 embryos in the presence of ssODN (Fig. 1B). This enrichment of the expected peak in the presence of repair template indicates that knock-in by HDR is occurring in some of the embryos. To validate that these expected size peaks were in fact due to integration of the ssODN, we cloned the gel purified PCR product from a positive embryo and sequenced 20 clones. Sequence analysis showed 5 clones with expected knock-in sequence as well as clean and precise integration at both ends of the ssODN (Additional file 1: Figure S2B). The remaining 15 clones were either WT (8 clones) or with random indels (7 clones) as expected due to the mosaicism for CRISPR-induced mutations in the embryo being analyzed. These data demonstrated that using CRISPR-STAT to look for an enrichment of the expected size peak in the embryos co-injected with the repair template compared to the sgRNA/Cas9 alone can be used for a quick evaluation of the sgRNA and ssODN designed for a knock-in experiment.

Previous studies have shown that preselection of founder fish by screening for knock-in allele in somatic tissue using fin biopsies allows for efficient germline transmission screening [23, 38, 39]. Therefore, to prioritize founders for germline screening, we performed CRISPR-STAT on fin biopsies of all adult founders (*n* = 133) and identified 13 founders with the expected knock-in size peaks (Table 1). After screening the progeny of 12 out of these 13 founders as pooled embryos, we identified 2 founders that transmitted the knock-in allele to their progeny (Table 1). These two founder fish were outcrossed again to confirm knock-in by screening of individual embryos by fluorescent PCR and sequencing (Fig. 1C, Additional file 1: Figure S2C, Table 1). Progenies from these two founder fish were grown to adulthood and heterozygous adults were identified by fin biopsies. Expression of FLAG tag was confirmed in 5 dpf *tcnba*^*FLAG/FLAG*^ embryos by RT-PCR (Fig. 1D, Additional file 2: Fig. 1 – Supporting data) and sequencing (Additional file 1: Figure S3). Thus, our CRISPR-STAT based screening of injected embryos and adult founder fin biopsies allowed us to successfully generate a stable line with FLAG tag at the 3’ end of *tcnba* while saving time, labor and costs associated with sequencing based screening methods.

**Table 1:** Summary of founder screening data

Gene	Somatic screening data		Germline transmission data			
	# of founder fish screened	# of founder fish positive	# of founder fish positive / # of founder fish screened	# of positive F1 pools / # of total F1 pools	# of positive F1 embryos / # of total F1 embryos	# of positive F1 adults / # of total F1 adults
*tcnba*	133	13	2/12	4/11	3/16 (18.8%)	9/45 (20.0%)
				9/16	2/16 (12.5%)	4/40 (10.0%)
*gata2b*	41	7	2/7	3/7	6/32 (18.8%)	26/96 (27.1%)
				4/24	2/58 (3.4%)	2/164 (1.2%)
*gba*	95	22	4/11 (1 precise, 3 with errors)	3/21	11/94 (11.7%)	10/96 (10.4%)

### Knock-in of HA tag at 3’ end of *gata2b*

**Fig. 2:**
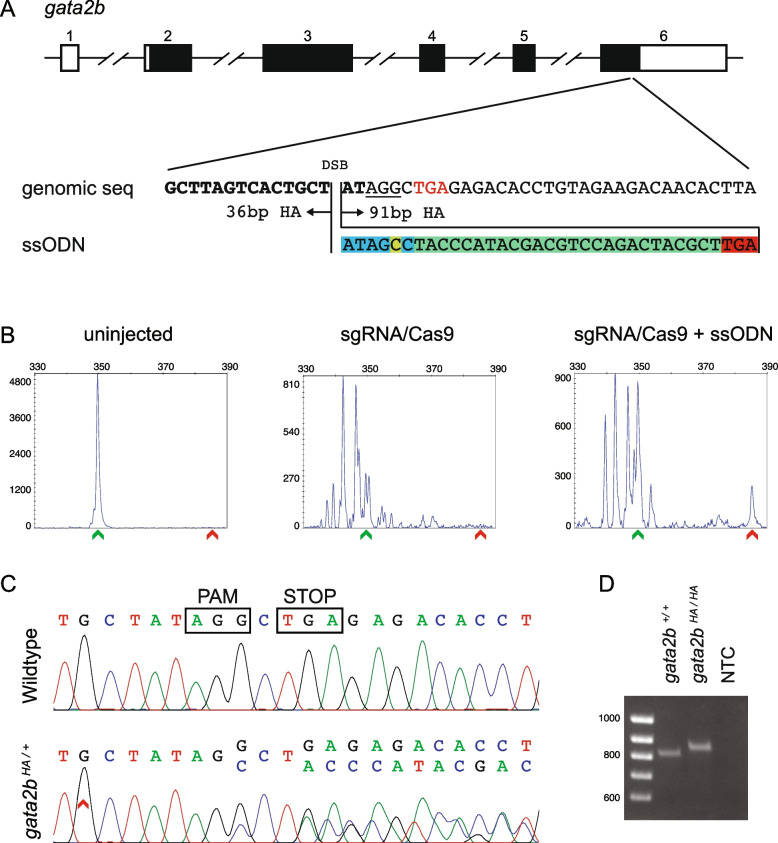
Design, screening, and validation of knock-in of HA tag at 3’ end of *gata2b*. **A** Schematic of *gata2b* genomic structure with coding region shaded in black (top panel), alignment of genomic sequence and ssODN template with direction of 36 bp and 91 bp homology arms (HA) marked. The ssODN contains a spacer (highlighted in blue) to maintain the ORF of *gata2b* as the double stranded break occurs 6 bp upstream of the stop codon (TGA, marked in red), a modification to the PAM site (G > C, highlighted in yellow) to prevent recutting after integration and the HA tag to be inserted (highlighted in green). **B** Representative CRISPR-STAT plots of uninjected, sgRNA/Cas9, and sgRNA/Cas9 plus ssODN injected embryos with X-axis showing the size of the peaks, Y-axis showing the peak height, and the size of the expected WT allele denoted by a green arrowhead. These plots were quantified for the presence of a peak generated by the insertion of ssODN (denoted by red arrowhead). **C** Sequence chromatograms showing WT and a positive F1 embryo to confirm germline transmission of knock-in sequence. **D** An agarose gel image showing expression of HA tag at *gata2b* locus by RT-PCR. RNA from *gata2b*^+*/*+^ or *gata2b*^*HA/HA*^ embryos were used as template and water was used as the no template control (NTC). The original gel image is provided in additional file 2: Fig. 2 – Supporting data and its cropped version is shown here

To evaluate our screening approach at another locus, we decided to add an HA tag to the 3’ end of *gata2b*, a transcription factor with critical roles in definitive hematopoiesis [40–42]. Two sgRNAs in close proximity of the stop codon were evaluated by CRISPR-STAT and sgRNA-T1 was selected (Additional file 1: Figure S4A-B). ssODN for repair template was designed based on this sgRNA and 6 bp between the DSB site and the stop codon were incorporated in front of the HA tag with an additional nucleotide change (G > C) to disrupt the PAM site (Fig. 2A, Additional file 1: Figure S5A). The expected knock-in peak, which is 36 bp larger than the WT peak, was detected in 4 out of 40 embryos in the presence of the repair template as opposed to only 1 in 31 of control embryos injected with sgRNA/Cas9 alone (Fig. 2B). Validation by cloning and sequencing of the PCR product from a positive embryo revealed correct knock-in of the ssODN with clean and precise integration at each end in 6/46 clones (Additional file 1: Figure S5B). Among the remaining clones, 33 clones contained indels and 7 clones were WT.

Pre-screening of adult founders by fin biopsies led us to prioritize 7 of the 41 fish for germline screening (Table 1). Multiple pools of embryos from 2 of these 7 founders showed expected knock-in peak (Table 1). These 2 founders were bred again, and individual embryos were screened by fluorescent PCR (Additional file 1: Figure S5C, Table 1) followed by sequence confirmation (Fig. 2C). Our data showed that these 2 founders transmitted the knock-in allele to 1.2 – 3.4% and 18.8 – 27.1% of their progeny (Table 1). After establishing a stable *gata2b*^*HA/HA*^ line, we confirmed the expression of the HA tag in 1 h post fertilization (hpf) *gata2b*^*HA/HA*^ embryos by RT-PCR (Fig. 2D, Additional file 2: Fig. 2 – Supporting data) and sequencing (Additional file 1: Figure S6). Based on these two examples, we believe that our screening strategy can be easily applied to insert a variety of small sequences, such as epitope tags or loxP sites at the desired sites in the genome.

### Experimental design for knock-in of point mutation D430H in the *gba* gene

**Fig. 3:**
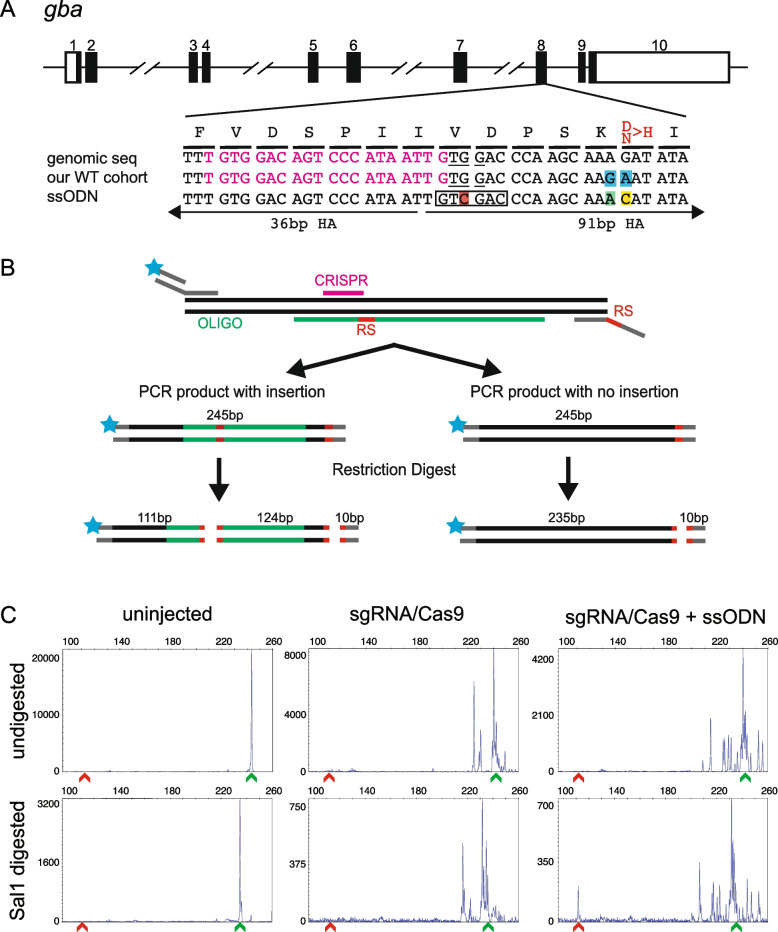
Design of ssODN and screening strategy for knock-in of a point mutation in *gba*. **A** Schematic of *gba* genomic structure with coding region shaded in black (top panel), alignment of genomic sequence, sequence in our cohort of fish showing 2 polymorphisms (highlighted in blue) and ssODN design with direction of 36 bp and 91 bp homology arms (HA) marked. Amino acids coded by each triplet are marked above the genomic sequence, with the amino acid to be changed marked in red. The ssODN contains a modification to the PAM site (G > C, highlighted in red) which creates a SalI restriction site (marked by black rectangle), a G > A modification to change the SNP in our WT cohort back to the reference sequence (highlighted in green) and the desired point mutation A > C (highlighted in yellow). **B** A schematic of the strategy for combining fluorescent PCR and RFLP analysis to detect integration of ssODN. Top panel shows the 3-primer fluorescent PCR strategy with fluorophore denoted by blue star and restriction sites marked as RS. Middle panel shows the two possible outcomes in injected embryos with size of the PCR product and location of either 2 (insertion of ssODN) or 1 (no insertion of ssODN) restriction sites (marked in red). The bottom panel shows the expected fragment sizes after the enzymatic digestion of the PCR product. Since only the fluorescently labelled fragments will be detected, embryos with knock-in can be identified by the presence of a 111 bp fragment. Multiple peaks around the WT size of 235 bp are expected due to CRISPR-induced random indels. C Representative CRISPR-STAT plots of uninjected, sgRNA/Cas9, and sgRNA/Cas9 plus ssODN injected embryos before and after SalI digestion with X-axis showing the size of the peaks, Y-axis showing the peak height, and the size of the expected WT allele denoted by a green arrowhead. Successful integration following digest results in a peak at 111 bp (denoted by red arrowhead)

It is estimated that ~ 60% of disease-associated human genetic variants are point mutations [43] and knockout mutants do not always recapitulate their effect on the gene function as not all point mutations lead to loss-of-function of the mutated gene. Therefore, zebrafish models with exact point mutations as seen in the patients are needed for their functional evaluation. Here, we present our CRISPR-STAT based screening strategy for introduction of targeted point mutations in zebrafish using HDR. To demonstrate our screening approach, we chose to develop a zebrafish model with a specific point mutation observed in patients with Gaucher disease, type 3c (GD3c) in the glucocerebrosidase gene (*GBA*) [44]. These patients are homozygous for D409H mutation and manifest specific ophthalmic phenotypes including an oculomotor abnormality consisting of slowed horizonal saccadic eye movements and corneal opacities [45]. However, the etiology of these ophthalmic manifestations is not well understood [46] and zebrafish offer well-established methods to study these phenotypes [47–49]. Human GBA D409 is equivalent to D430 in the zebrafish Gba and is located in exon 8 of *gba* gene (Fig. 3A). Sequencing of this region in our WT strain showed two single nucleotide polymorphisms (SNPs) when compared to the reference sequence (GenBank accession XM_682379); an A > G change (silent) and a G > A change (D430N) (Fig. 3A). Therefore, we designed the ssODN against the sequence of our cohort of WT fish. The most efficient sgRNA generated a DSB 17 bp upstream from the target nucleotide (Fig. 3, Additional file 1: Figure S7A-B). The ssODN was designed with the following three modifications: 1) A > C to generate the intended point mutation (N430H in our cohort), 2) G > A to change the endogenous SNP back to the reference genome sequence and 3) a silent change (G > C) in the PAM site to act as a CRISPR/Cas-blocking mutation (Fig. 3A, Additional file 1: Figure S8A).

Since our strategy for detecting knock-in alleles with insertion of epitope tags relied on the change in size of the PCR product, it would not work for detection of point mutations as there is no change in the size of the PCR product. Thus, we combined CRISPR-STAT with RFLP analysis by adding a restriction site to the ssODN (Fig. 3B) to distinguish the knock-in allele from the WT allele. In this example, the silent change in PAM site created a new SalI restriction site (Fig. 3A) which leads to a digested fragment size of 111 bp if the ssODN is inserted (Fig. 3B). To avoid false negatives due to restriction digest failure, we added the same restriction site to the tail of reverse primer (Table S1) to serve as an internal control for restriction enzyme digestion as it leads to the removal of last 10 bp from the PCR product (Fig. 3B). Analysis of WT (uninjected), sgRNA/Cas9, and sgRNA/Cas9 plus ssODN injected embryos showed 111 bp peak in the latter group of embryos (12/60) after digestion with SalI (Fig. 3C). TOPO cloning and sequencing of 53 clones from a positive embryo confirmed correct knock-in of the ssODN with all three of the introduced nucleotide changes and clean integration at each end in one clone (Additional file 1: Figure S8B). The remaining 52 clones were either WT (18 clones) or contained indels (34 clones).

**Fig. 4:**
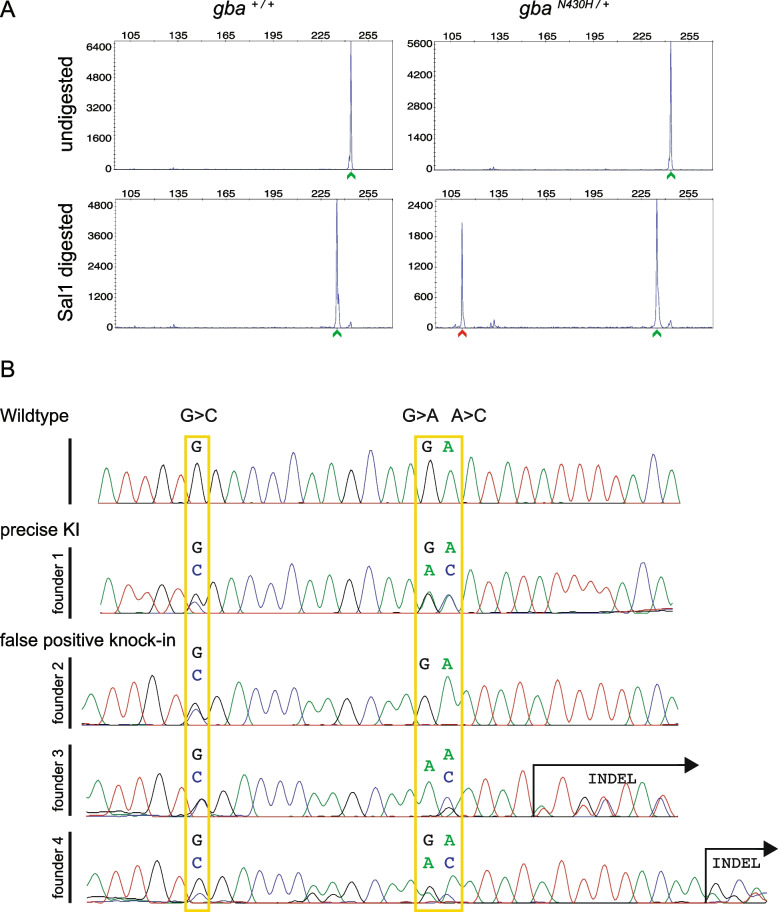
Germline screening data for knock-in of the point mutation in *gba*. **A** Representative plots of undigested and SalI digested fluorescent PCR products showing detection of the mutant allele (N430H) denoted by the red arrowhead in the progeny of a germline transmitting founder. In all plots, the X-axis represents the size of the peaks and the Y-axis shows the peak height. WT alleles (denoted by green arrowhead) are slightly smaller after digest (bottom panel) due to the restriction site in reverse primer used as internal control for successful restriction enzyme digestion. **B** Sequence chromatograms from representative embryos from each of the 4 founders positive for somatic knock-in compared with a WT embryo. Expected nucleotide modifications by knock-in of the ssODN are marked by yellow rectangles. Only founder 1 transmitted precise integration of the ssODN with all 3 modifications while the other 3 founders were false positives. Founder 2 had incomplete integration (the nucleotide for the restriction site was modified (G > C) but the desired (A > C) allele not changed) and founders 3 and 4 had indels

Data from pre-screening of adult founders and germline transmission screening using the SalI digest followed by CRISPR-STAT analysis are shown in Table 1. Of the 22 prioritized founder fish, we identified four positive founders after screening pooled embryos from 11 founders and therefore, the remaining 11 founders were not screened (Table 1, Fig. 4A). Sequencing showed that progeny of only 1 founder had precise integration of the ssODN with a germline transmission rate of 10.4 – 11.7% (Fig. 4B, Table 1). Among the remaining three founders, founder 2 transmitted knock-in of the restriction site but not the desired point mutation (Fig. 4B). We speculate that due to the large distance between the cut site and the desired point mutation (17 bp), replicative repair occurred within this region as has been previously demonstrated [50] and thus only the PAM site change was transmitted to the progeny of this founder. Founders 3 and 4 each transmitted the restriction site and desired allele but also contained indels (Fig. 4B). Thus, a limitation of our screening method is that false positives due to imprecise knock-in either due to replicative repair occurring between the restriction site and the point mutation or due to indels at the 3’ end of the PCR product will be identified since only 5’ end of the PCR product is detected after digest. Hence, we recommend identifying multiple germline transmitting founders and validate by sequencing to identify one with precise knock-in at both ends. With this example, we have demonstrated that CRISPR-STAT can be easily adapted with the addition of a restriction digest to detect knock-in of point mutations.

### Proposed knock-in pipeline using our screening methods

**Fig. 5:**
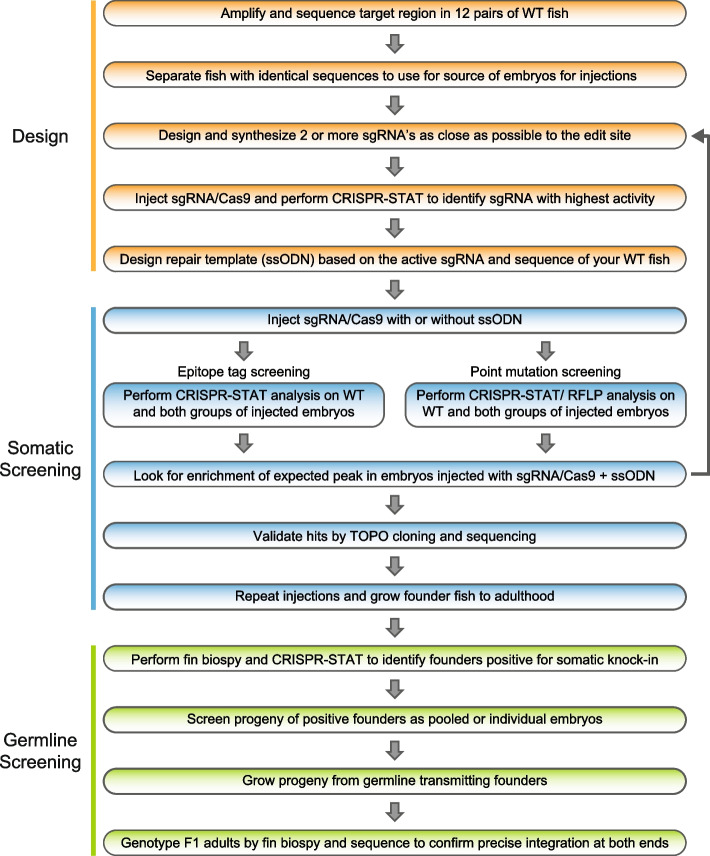
Workflow of our 3-phase knock-in pipeline. The design phase consists of five steps involving selection of WT fish for injections by sequence analysis of the target region in your WT fish line, finding an active sgRNA and designing the ssODN based on these data. The somatic screening phase occurs following injections. Embryos are collected and CRISPR-STAT or CRISPR-STAT/RFLP analysis is performed to determine if there is an enrichment of the expected peak in the sgRNA/Cas9 + ssODN group. If no enrichment is seen, another sgRNA/ssODN combination needs to be designed and tested. If expected size peaks are observed, representative samples are TOPO cloned and sequenced to confirm knock-in. The last phase is germline screening and begins by pre-screening of adult founder fish by fin biopsies for somatic knock-in. Positive fish are then prioritized for breeding to screen for germline transmission. Founders that transmit knock-in allele to their progeny are bred to grow F1 adults for genotyping, and sequence confirmation of precise knock-in

Based on the successful generation of knock-in fish lines at three different loci, we propose to use our pipeline as illustrated in Fig. 5 to introduce point mutations or insert small sequences at desired loci using ssODNs. This pipeline consists of 3 phases: Design, somatic screening, and germline screening. The design phase takes about 2 weeks and is the most important step as it ensures that the chosen sgRNA and repair template combination would lead to successful knock-in. The major considerations in the design phase are to sequence your cohort of WT fish to identify fish with 100% homology [36, 51], find highly active sgRNAs and use these as the basis for design of the ssODN template for HDR. Somatic screening occurs after injections and is used to look for an enrichment for expected size peak in the presence of ssODN. This step takes only about a week and lets one to decide whether to proceed with growing injected embryos for founder screening or redesign a new sgRNA/ ssODN combination. During the germline screening phase, founder fish are first screened by fin biopsies to select fish positive for expected knock-in peak and thus most likely to transmit to their progeny. These prioritized fish are then outcrossed and their progeny is screened to identify the germline transmitting founders. This step takes about 2–3 weeks. Embryos from germline transmitting founders are then grown to adulthood (~ 3 months) and these F1 adults are genotyped by fin biopsy. All positive F1 adult fish are then sequenced thorough the entire ssODN to confirm precise integrations at both ends. Overall, use of this pipeline allowed us to generate knock-in alleles quickly and efficiently for our research and should prove useful to the entire zebrafish community.

## Discussion

Despite recent advances in genome editing tools, the highly inefficient process of HDR still presents a major hurdle in developing zebrafish models with targeted knock-in of desired sequences. Thus, there is a need for efficient screening methods to identify rare knock-in events, specifically when inserting small sequences with ssODNs due to the lack of visual screening tools. In this study, we developed a robust pipeline using fluorescent PCR and CRISPR-STAT based novel screening methods for quick, reliable, and cost-effective generation of fish lines with desired point mutations and small insertions using ssODNs as the repair template. We have demonstrated the efficiency of our screening methods with successful generation of fish lines with insertion of epitope tags at 2 loci and a patient specific point mutation at the third locus. For insertion of epitope tags, we took advantage of knowing the exact size of the knock-in allele compared to the WT allele. For knock-in of point mutations, we used restriction digest to generate fragments of different sizes based on if the knock-in occurred or not. In the example presented here, a silent nucleotide change in the PAM site added to prevent recutting also led to a new SalI restriction site. Alternately, other silent changes can be introduced in the ssODN to create a new restriction site. In all 3 examples, we used the enrichment for expected size fragment in injected embryos as an indication of knock-in and successfully identified germline transmitting founders by looking for the expected size peak in their fin biopsies. Consistent with previous studies [23, 38, 39], we found that the prioritization of founder fish by pre-screening reduced the number of fish to be outcrossed for germline screening. In total, pre-screening of founder fish eliminated the need to screen 239 fish and allowed recovery of precise knock-in alleles for all 3 loci by screening a total of 30 fish with germline transmission rates ranging from 1.2–27.1%.

Our methods present several advantages over the commonly used sequencing-based screening approaches. First, our methods are based on fluorescent PCR [6] and CRISPR-STAT [34], both of which are becoming popular in the zebrafish community for accurate genotyping of fish with indels and evaluation of sgRNAs, respectively [21, 52–60]. Thus, it would be easy to implement them for knock-in screening as described here. Second, these methods allow one to determine if knock-in is occurring in injected embryos within a week, thus saving considerable amount of time, fish facility space and husbandry costs associated with growing fish if no knock-in occurred. Third, due to the single base pair resolution provided by capillary electrophoresis, false positives due to imprecise integrations are expected to be rare. We did not observe any false positive founders in epitope tag insertions. However, we did observe false positives in screening for point mutation, specifically indels at the 3’ end of the ssODN were observed in 2 out of 4 founders. This is expected as our method relies on the restriction site which is present even in cases of knock-in occurring with simultaneous indels. While indels at the 5’ end of ssODN integration site would lead to a change in the size of the digested fragment, indels at the 3’ end are missed as it gets cleaved off after the digest. However, unlike allele-specific PCR [24] or NGS based screening methods [11, 17, 21, 24, 60] that require additional validation steps, our primers are designed to not overlap with the ssODN and therefore, we could simply sequence the PCR product to confirm precise or imprecise integration events without additional amplification steps. Thus, although there is a caveat to our screening approach for point mutations, it can be overcome by screening additional prioritized founders and sequence validation. As demonstrated previously [34], CRISPR-STAT is sensitive enough to detect up to 20% mosaicism. Therefore, it is possible to miss low level knock-in events during somatic screening, especially after restriction digest. However, we believe that this limitation can be overcome by screening additional prioritized founder fish for germline transmission to establish the desired knock-in fish model.

The best way to put our screening approach in perspective with other commonly used knock-in screening methods, i.e., PCR or PCR/RFLP analysis by gel electrophoresis, AS-PCR and NGS is to compare our founder screening efficiencies with those published using these methods [16, 17, 19, 21, 22, 24, 38]. Since the knock-in efficiency can be influenced by several factors, i.e., design of sgRNA, the proximity of the cut site to the insertion site, and design of the donor template, we selected two studies for comparison where donor design criteria used were similar to ours [21, 24]. Their recovery rate of founders transmitting precise knock-in events ranged from 3.3–21.4% by AS-PCR [24] and 10.0–18.2% by NGS [21]. In our study, following preselection of founders by fin biopsies, we were able to identify precise knock-in in 9.1–28.6% of founders screened for germline transmission. Overall, our screening method performed at comparable or higher levels than NGS and AS-PCR and provides an alternate option between costly but high throughput methods (NGS) and cheaper and easily accessible but low throughput methods (AS-PCR or PCR/RFLP assay) for screening to establish desired zebrafish models with knock-in using ssODNs. To overcome the limitation of access to a capillary electrophoresis machine, commercial vendors or Institute’s sequencing cores can be used for fragment size analysis.

## Conclusions

In this study, we have established a robust pipeline to efficiently generate fish with small DNA insertions as well as point mutations (Fig. 5). By using capillary electrophoresis, we can detect low frequency events in somatic tissue to quickly determine the success of knock-in design. To detect point mutations, we developed a CRISPR-STAT/RFLP hybrid assay that was highly successful in generating fish lines without having to sequence an exorbitant number of samples. We believe that these methods would be of tremendous help to zebrafish researchers in generating their desired fish models using knock-in for insertion of small sequences or nucleotide substitutions. Given the versatility of fluorescent PCR and CRISPR-STAT, these methods could easily be scaled or adapted by researchers using a variety of model systems to efficiently screen and detect knock-in alleles.

## Methods

### Zebrafish husbandry

Zebrafish (*Danio rerio*) from WT strain TAB-5 [61], generated in Hopkins lab at Massachusetts Institute of Technology and maintained in our own aquatic facility for several years, were used in this study. All zebrafish experiments were done under an approved animal study protocol. Embryo care and zebrafish handling were performed as described in the Zebrafish book [62]. Adult male and female zebrafish were bred to obtain embryos, which were raised in an incubator at 28.5ºC until 5–6 dpf. Larvae and adult animals were housed in a recirculating aquatic system with a light–dark cycle (14/10 h) and a water temperature of 28ºC. The final number of zebrafish used in this study was 2400 (1600 embryos, 800 adults). Number of fish used in each of the three projects described here was determined based on the expected viability of injected embryos grown to adulthood (~ 80%) and expected efficiency of achieving desired knock-in events (< 10%) without a priori power calculations. Anesthesia and euthanasia were performed as required using approved animal care and use committee guidelines. Anesthesia was performed on adult animals prior to fin biopsy by submersion in 0.4 g/L MS-222 (Western Chemical) buffered to pH 7. Following fin biopsy, animals were allowed to recover in aquatic water in a tank before being placed back onto the housing system. Embryos (≤ 5 dpf) were euthanized by rapid freezing in a -80ºC freezer followed by immersion in lysis buffer.

## sgRNA and Cas9 mRNA synthesis and microinjections

CRISPR target sites (Table S1) were identified using the CRISPRscan [63] or ZebrafishGenomics [7] tracks on assembly GRCz11/danRer11 in the UCSC Genome Browser. sgRNAs and Cas9 mRNA were prepared using previously described protocols [13]. WT embryos were injected at the 1-cell stage using a PicoPump (World Precision Instruments) and standard microinjection protocols [62]. Injection mixes contained 300 pg Cas9 mRNA and 50 pg sgRNA with or without 25 pg of ssODN template.

### DNA Extraction and CRISPR-STAT for evaluation of sgRNA activity

Extraction of DNA and CRISPR-STAT were performed as previously described [13]. Briefly, embryos were collected at 1 dpf and euthanized for DNA extraction using the Sigma Extract-N-Amp kit. Fluorescent PCR amplification was then performed using equimolar ratios of forward, reverse and a universal 6FAM-M13F primer with the following conditions: 12 min denaturation at 94 °C; 40 cycles of 94 °C for 30 s, 57 °C for 30 s, and 72 °C for 30 s; and final extension at 72 °C for 10 min followed by a hold at 4 °C. Sequences of all primers used for fluorescent PCR are listed in Table S1. PCR products were then mixed with 1:50 mix of GeneScan 400HD ROX dye size standard and Hi-Di Formamide (ThermoFisher) and run on a 3130xl or a 3730-sequencer using CRISPR-STAT settings (double injection time). Data analysis was performed using GeneMapper (ThermoFisher) to determine activity for each sgRNA.

### Design of ssODNs and screening primers

Fin biopsies from anesthetized WT fish were sequenced using primers listed in Table S1 to identify a cohort of fish with identical sequences in the target region. Asymmetric ssODNs were designed based on the active sgRNA and sequence of our cohort of WT fish. We followed the guidelines from Richardson and colleagues [64] to design ssODNs against the nontargeting strand with the sequence to be inserted flanked by a 36 bp homology arm on the distal side and 91 bp homology arm on the proximal side of the DSB. In addition, ssODNs were designed with PAM site modifications known as CRISPR/Cas9-blocking mutations [65] to prevent recutting after HDR. All ssODNs were synthesized as Ultramer DNA oligos (Integrated DNA technologies). Screening primers were designed from the genomic regions outside of the homology arms to prevent false positives that can result from imprecise integration of the repair template (Table S1).

## Somatic screening for knock-in of epitope tags

At 1 dpf individual uninjected control (*n* = 8), sgRNA/Cas9-injected (*n* = 16) and sgRNA/Cas9 plus ssODN-injected embryos (*n* = 24) were collected, euthanized, and processed for DNA extraction, fluorescent PCR and capillary electrophoresis as described above. These numbers allowed us to analyze embryos from all desired combinations in half of a 96 well plate. Plots for each embryo were analyzed and scored in GeneMapper (ThermoFisher) for the presence or absence of peaks at the expected knock-in size. If a peak was detected above the background threshold, the sample was considered positive. After positive hits were detected and confirmed by cloning as described below, the remaining injected embryos were grown to adults for germline screening. If additional embryos needed to be injected to grow the founder generation, somatic analysis was repeated to rule out any technical issues with injections.

### Somatic screening for knock-in of point mutations

At 1 dpf individual uninjected control (*n* = 8), sgRNA/Cas9-injected (*n* = 16) and sgRNA/Cas9 plus ssODN-injected embryos (*n* = 24) were collected in a half a 96 well plate, euthanized, and processed for DNA extraction and fluorescent PCR as described above. Five µl of PCR product was mixed with 0.2 µl (4U) SalI-HF enzyme, 1 µl cut smart buffer (New England BioLabs) and 3.8 µl water and incubated at 37 °C for 1.5 h followed by inactivation at 65 °C for 20 min. Samples were then mixed with GeneScan 400HD ROX dye size standard/ Hi-Di Formamide and run as previously described. Scoring of plots and subsequent steps of confirmation of positive hits by cloning and growing injected embryos to adulthood were performed as described above for epitope tags.

### Cloning and sequencing of positive samples

To confirm knock-in in injected embryos, we repeated PCR from a representative CRISPR-STAT positive sample for each target site without the 6FAM-M13F primer. PCR products from *gata2b* and *gba* samples were purified using a MinElute PCR purification kit (Qiagen). PCR products from *tcnba* samples were gel purified using the QIAquick gel extraction kit (Qiagen) to specifically enrich for knock-in product due to its relatively lower intensity compared to the other peaks. Purified PCR products were cloned in pCR4-TOPO vector (ThermoFisher), and colony PCR was performed on all colonies with the same set of primers used to amplify the original product. Colony PCR products were treated with ExoSAP-IT (ThermoFisher) and sequenced using BigDye Terminator v3.1 (ThermoFisher). Sequences were analyzed using Sequencher (Gene Codes) to determine the number of clones with desired knock-in.

### Prioritization of founders and germline screening to establish stable lines

All founder fish were pre-screened by CRISPR-STAT (epitope tags) or CRISPR-STAT/RFLP (point mutations) analysis of their fin biopsies. Founder fish with positive hits for the expected peak were then used in pairwise outcrosses with WT fish. Four founders could be screened on a single 96 well plate by collecting embryos at 1 dpf in pools of 3 embryos/well (up to 24 pools/founder), euthanized, and processed for DNA extraction and fluorescent PCR followed by SalI digestion for *gba* samples. All samples were then run on a sequencer with standard fluorescent PCR settings [13] and the plots were analyzed in GeneMapper (ThermoFisher) for the expected knock-in allele peak. The founders with positive hits in the pooled embryos were outcrossed again to screen up to 96 individual embryos, followed by sequencing of representative positive embryos for confirmation of precise knock-in. To establish stable lines, embryos from germline transmitting founders were grown to adults and genotyped by fluorescent PCR.

### RT-PCR to confirm expression of epitope tags

Embryos homozygous for the knock-in alleles were euthanized and collected at different time points depending upon when the corresponding gene is known to be expressed at high level: for *tcnba* (5 dpf) [37] and for *gata2b* (1 hpf) [42]. RNA was extracted from these embryos along with the age-matched WT embryos using the Direct-zol RNA Microprep kit (Zymo Research). RT-PCR was performed using primers listed in Table S1 and the SuperScript III One-Step RT-PCR System (ThermoFisher) with the following conditions: 50 °C for 30 min, 94 °C for 2 min; 40 cycles of 94 °C for 15 s, 57 °C for 30 s, 72 °C for 1.5 min; 72 °C for 10 min. RT-PCR products were analyzed by gel electrophoresis using 1.5% agarose gels, imaged with an Azure Biosystem 200 imaging system and sequenced as described above to confirm expression.

## Supplementary Information

**Additional file 1:** **Table S1. **Sequences of all primers and sgRNA's used in this study. **Figure S1.** *tcnba* CRISPR selection and CRISPR-STAT analysis. **A** Screenshot of the *tcnba* target region from UCSC genome browser using the CRISPRscan track showing possible sgRNAs near the stop codon. **B** Evaluation of individual embryos by CRISPR-STAT to determine activity levels. In all plots, the X-axis shows the size of the peaks and the Y-axis shows the peak height. sgRNA-T2 showed higher activity and was selected for knock-in experiments. **Figure S2.** Design of *tcnba* ssODN and somatic and germline screening. **A** Detailed design of the ssODN aligned with the ref sequence (chr5:30,618,013-30,618,151) and sgRNA used for generating the DSB. **B** Sequence chromatogram of a clone from an injected embryo positive for the knock-in peak confirming the insertion of the desired sequence as well clean and precise integration at each end of the ssODN. **C** Representative plot (X-axis showing the size of the peaks and the Y-axis showing the peak height) from an F1 embryo heterozygous for the knock-in allele (denoted by red arrowhead) compared to a WT embryo. **Figure S3.** Sequence confirmation of the *tcnba*^*FLAG/FLAG*^ RT-PCR product. **Figure S4.** *gata2b* CRISPR selection and CRISPR-STAT analysis. **A** Screenshot of UCSC genome browser using the CRISPRscan and ZebrafishGenomics tracks showing possible sgRNAs near the stop codon. **B** Evaluation of individual embryos by CRISPR-STAT to determine activity levels. In all plots, the X-axis shows the size of the peaks and the Y-axis shows the peak height. sgRNA-T1 showed higher activity and was selected for knock-in experiments. **Figure S5.** Design of *gata2b* ssODN and somatic and germline screening. **A** Detailed design of the ssODN aligned with the ref sequence (chr6:40,803,222-40,803,359) and sgRNA used for generating the DSB. **B** Sequence of a TOPO clone from an injected embryo positive for the knock-in peak confirming the insertion of the desired sequence as well clean and precise integration at each end of the ssODN. **C** Representative plot (X-axis showing the size of the peaks and the Y-axis showing the peak height) from an F1 embryo heterozygous for the knock-in allele (denoted by red arrowhead) compared to a WT embryo. **Figure S6.** Sequence confirmation of *gata2b*^*HA/HA*^ RT-PCR product. **Figure S7.** *gba* CRISPR selection and CRISPR-STAT analysis. **A** Screenshot of UCSC genome browser using the CRISPRscan track showing possible sgRNAs near the desired point mutation. **B** Evaluation of individual embryos by CRISPR-STAT to determine activity levels. In all plots, the X-axis shows the size of the peaks and the Y-axis shows the peak height. sgRNA-T1 showed higher activity and was selected for knock-in experiments. **Figure S8.** Design of *gba* ssODN and sequence confirmation of knock-in in injected embryos. **A** Detailed design of the ssODN aligned with the ref sequence (chr16:14,227,951-14,228,090), sequence of our WT cohort of fish, the sgRNA used for generating the DSB and the SalI restriction site that is generated by the ssODN. **B** Sequence of a TOPO clone from an injected embryos positive for the knock-in peak confirming the presence of the desired nucleotide changes as well clean and precise integration at each end of the ssODN.

**Additional file 2.** Original images for gels shown in Figures 1D and 2D.

## Data Availability

The sequences of genes targeted in this study were obtained from GenBank as follows: *tcnba* (Accession number NM_001128735), *gata2b* (Accession number NM_001002689) and *gba* (Accession number XM_682379). All Sanger sequencing data from TOPO cloning and founder screening are available as FASTQ files in the Sequence Read Archive of the National Center for Biotechnology Information database under BioProject PRJNA889809 (http://www.ncbi.nlm.nih.gov/bioproject/889809). Information about the three new zebrafish lines generated during this study is available at The Zebrafish Information Network as follows: tcnba-FLAG (hg132): https://zfin.org/ZDB-ALT-220104-1; gata2b-HA (hg133): https://zfin.org/ZDB-ALT-220104-2; gba-D430H (hg134): https://zfin.org/ZDB-ALT-220104-3.

## Abbreviations

AS-PCR: Allele-specific PCR
CRISPR: Clustered Regularly Interspaced Short Palindromic Repeats
CRISPR-STAT: CRISPR Somatic Tissue Activity Test
dpf: Days post fertilization
DSB: Double stranded break
HDR: Homology-directed repair
hpf: Hours post fertilization
ICE: Inference of CRISPR Edits
NGS: Next-generation sequencing
NHEJ: Non-homologous end joining
ORF: Open reading frame
SNPs: Single nucleotide polymorphisms
ssODNs: Single-stranded oligodeoxynucleotides
RFLP: Restriction fragment length polymorphism
TIDER: Tracking of Insertions, Deletions and Recombination events
WT: Wildtype
