# Supplementary material for: A robust pipeline for efficient knock-in of point mutations and epitope tags in zebrafish using fluorescent PCR based screening
Source: BMC Genomics. 2022 Dec 7;23:810. doi: 10.1186/s12864-022-08971-1 (PMC9730659; doi:10.1186/s12864-022-08971-1)

Table S1. Sequences of all primers and sgRNA's used in this study.

Purpose	Primer Name	Sequence	Size of WT/knock-in PCR product (bp)
Sequencing of target region	tcnba-E4-SNP-Fwd	TGTAAAACGACGGCCAGT TGAGGATCAGACATACTGGG	568
	tcnba-E4-SNP-Rev	CAGGAAACAGCTATGACC AGAGGTAACCGTGAGTGTGC	
	gata2b-E6-SNP-Fwd	TGTAAAACGACGGCCAGT AAGGATGGTATTCAGACACG	539
	gata2b-E6-SNP-Rev	CAGGAAACAGCTATGACC AGTTCCATAACAGTGCAGTCG	
	gba-E8-Fwd	TGTAAAACGACGGCCAGT ATTATGTGACCGGCTGGACC	358
	gba-E8-SNP-Rev	CAGGAAACAGCTATGACC GACGTCACACTTAACAAGCG	
Screening and sequence validation of knock-in	tcnba-E4-Fwd	TGTAAAACGACGGCCAGT TTGCAGGAATCGGCTGCTAC	333/376
	tcnba-E4-Rev	GTGTCTT AGAGGTAACCGTGAGTGTGC	
	gata2b-E6-Fwd	TGTAAAACGACGGCCAGT TGTGTGCACGACAAACCATCC	350/386
	gata2b-E6-Rev	GTGTCTT CAAAACTCTTGGTGTACTGCG	
	gba-E8-Fwd	Same as listed above	235/111 (sizes after digest)
	gba-E8-Sal1-Rev	GTGTCTT GTCGAC GGTGTTCTTGCACAAGATCTG	
RT-PCR	gata2b-E4-RT-Fwd	TGTAAAACGACGGCCAGT GTAAATTGTGGAGCTACGTCG	821/856
	gata2b-E6-RT-Rev	CAGGAAACAGCTATGACC AGTTCCATAACAGTGCAGTCG	
	tcnba-E2-RT-Fwd	ACTGTGATCCAGGAAGGC	326/369
	tcnba-E4-RT-Rev	GATGTGGTGTTAATTCAGCAG	
Universal primer for fluorescent PCR	M13-FAM	6FAM-TGTAAAACGACGGCCAGT	n/a
sgRNA and PAM sequences (red letters denotes where a G was substituted in order to use T7 for synthesis)	gata2b-sgRNA-T1	AGCAGCTTAGTCACTGCTAT (AGG)	n/a
	gata2b-sgRNA-T2	AGTAAGTGTTGTCTTCTAC (AGG)	
	tcnba-sgRNA-T1	GGCCCAAGTGGTGAACCTC (AGG)	
	tcnba-sgRNA-T2	GATCCTGAGGTTCACCACTT (GGG)	
	gba-sgRNA-T1	TGTGGACAGTCCCATAATTG (TGG)	
	gba-sgRNA-T2	TGTAAAATATATCTTTGCTT (GGG)	

Figure S1: *tcnba* CRISPR selection and CRISPR-STAT analysis.

A

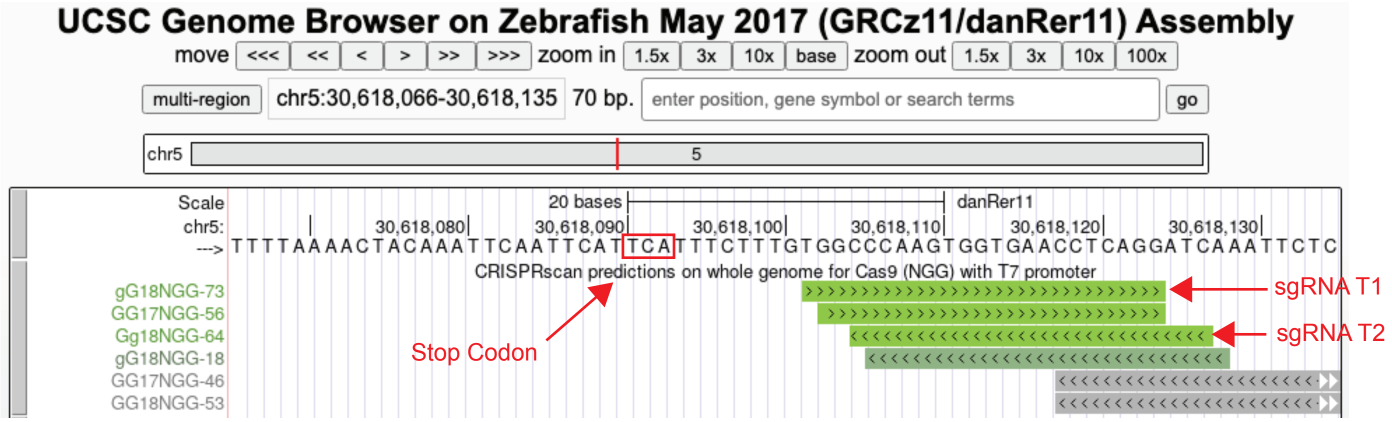

* *tcnba* on minus strand

B

CRISPR-STAT analysis to determine activity level for sgRNA’s

uninjected sgRNA-T1 sgRNA-T2

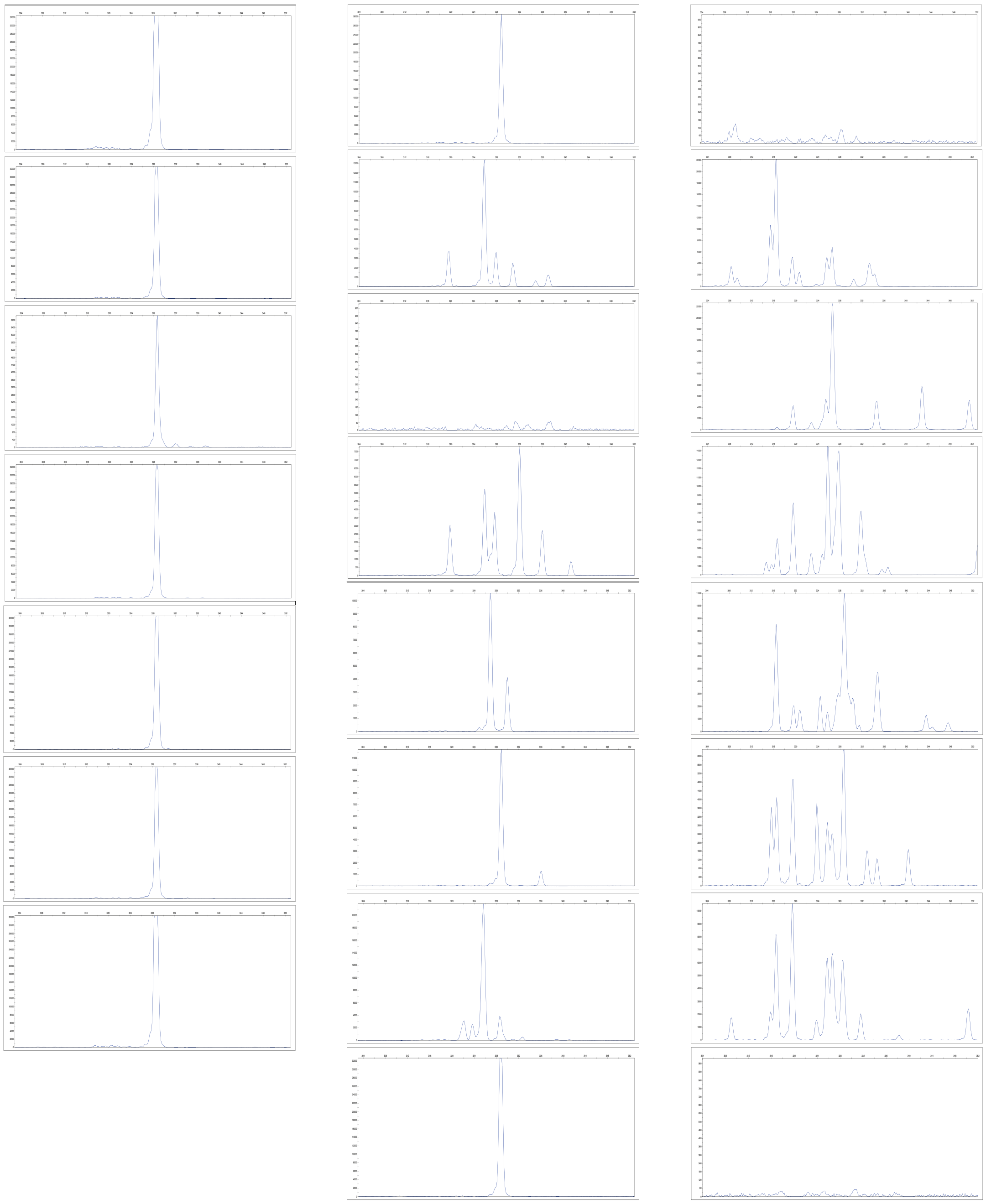

Figure S2: Design of *tcnba* ssODN and somatic and germline screening.

A. Detailed design of *tcnba* ssODN

```
ref seq   CCAAGTTCAGAGGGATGAGAATTTGATCCTGAGGTTCACCAC-------------------------------------------TTGGGGC
ssODN          TCAGAGGGATGAGAATTTGATCCTGAGGTTCACCACTTGGCCCACAAAGAAAGACTACAAAGACGATGACGACAAGTGATTGGGGC
sgRNA                             GATCCTGAGGTTCACCACTTGGG

ref seq   CACAAAGAAATGAATGAATTGAATTTGTAGTTTTAAAAGTTACTGCTGAATTAACACCACATCTTGTTTCAGTCATGTAATCAACAAGTTT
ssODN     CACAAAGAAATGAATGAATTGAATTTGTAGTTTTAAAAGTTACTGCTGAATTAACACCACATCTTGTTTCAGTCATGTAATCAAC
sgRNA
```

B. Sequence of TOPO clone showing clean inseriton of desired knock-in sequence

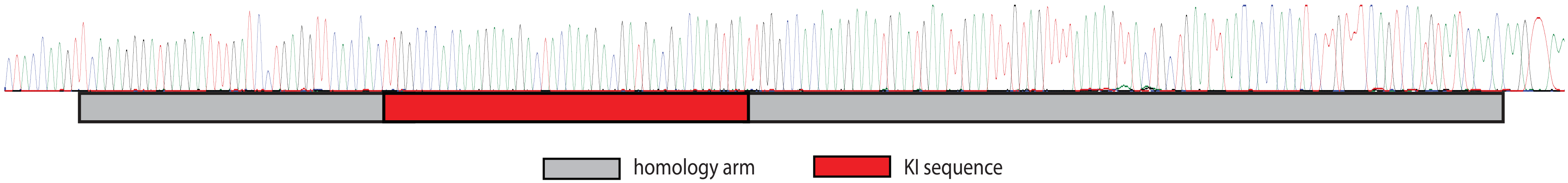

C. Representative plot from an F1 embryo heterozygous for the knock-in allele

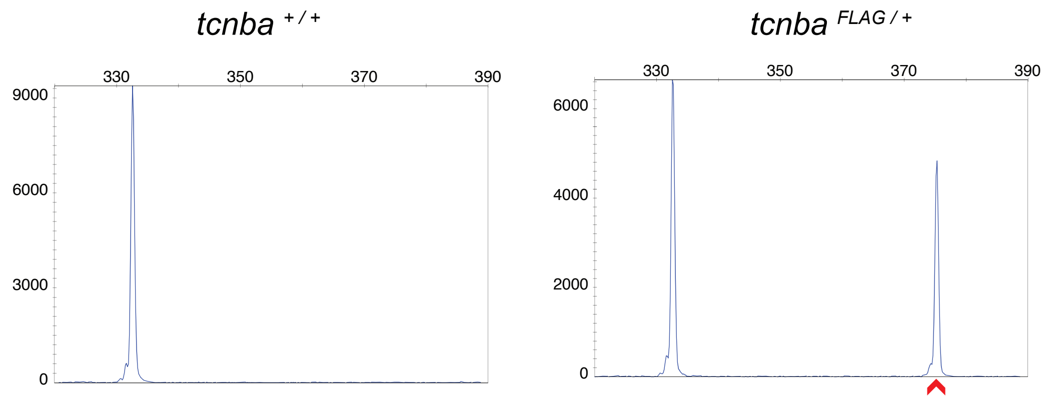

Figure S3: Sequence confirmation of *tcnba*$^{FLAG/FLAG}$ RT-PCR product.

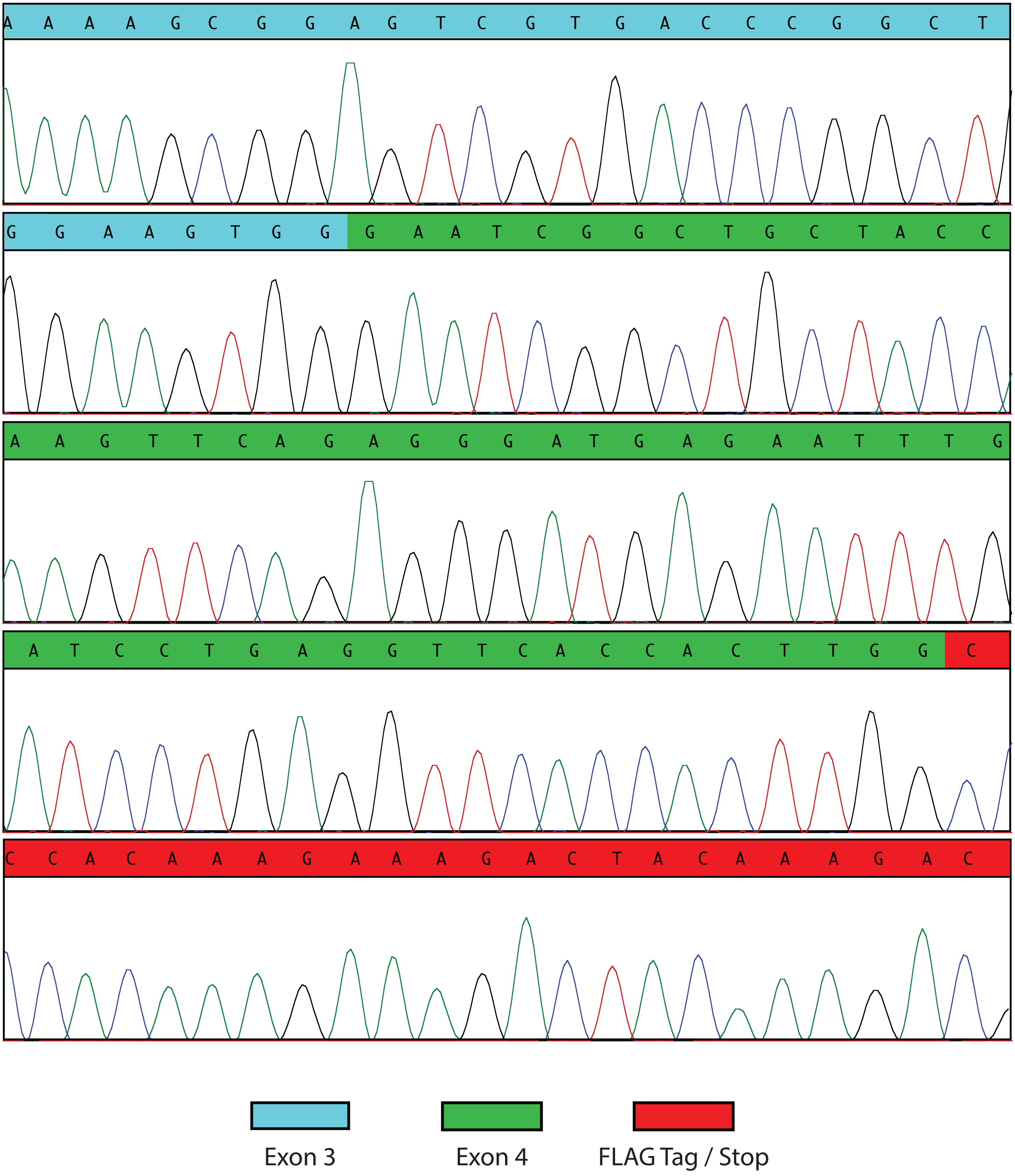

Figure S3 continued: Sequence confirmation of *tcnba*$^{FLAG/FLAG}$ RT-PCR product.

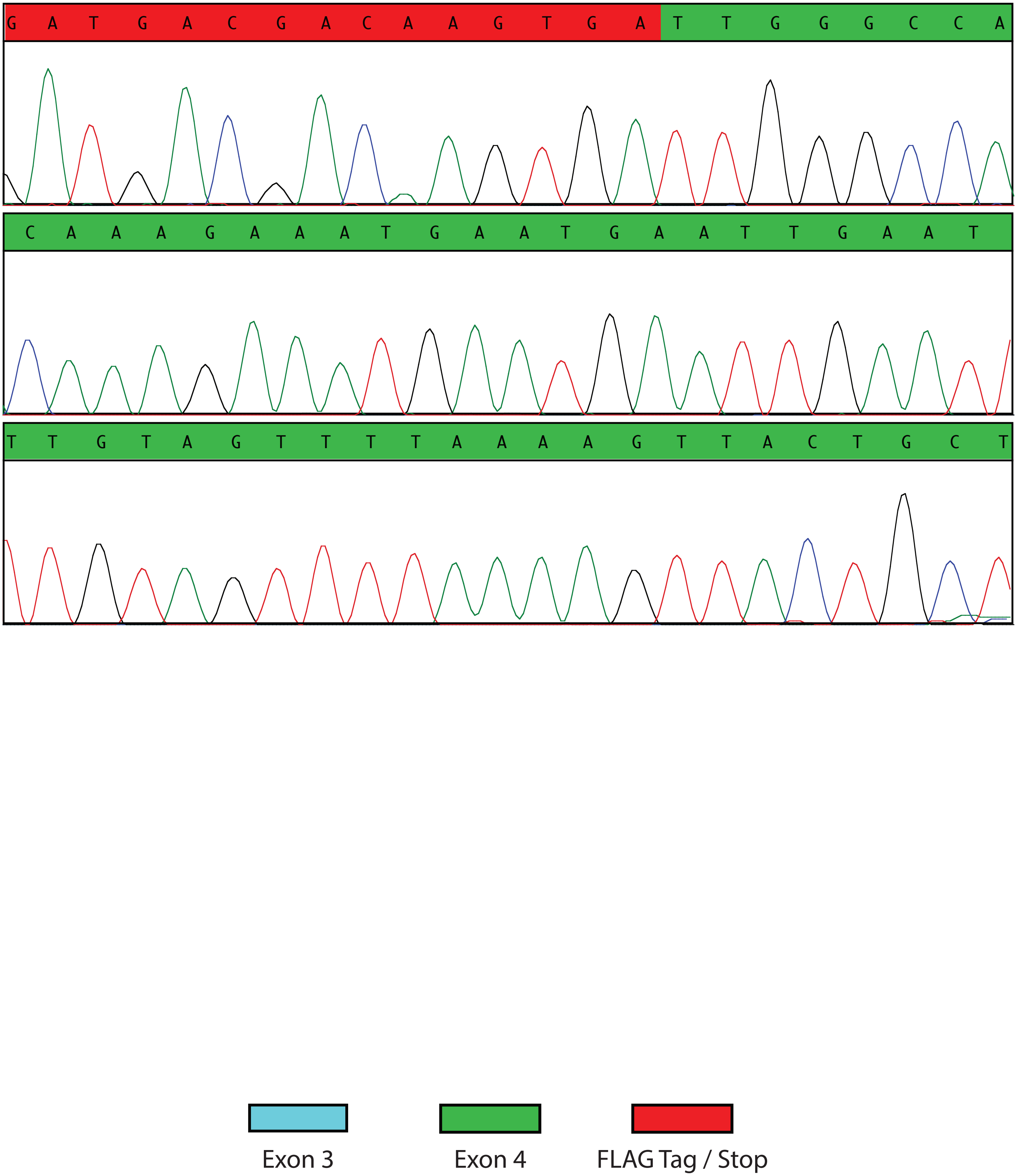

Figure S4: *gata2b* CRISPR selection and CRISPR-STAT analysis.

A

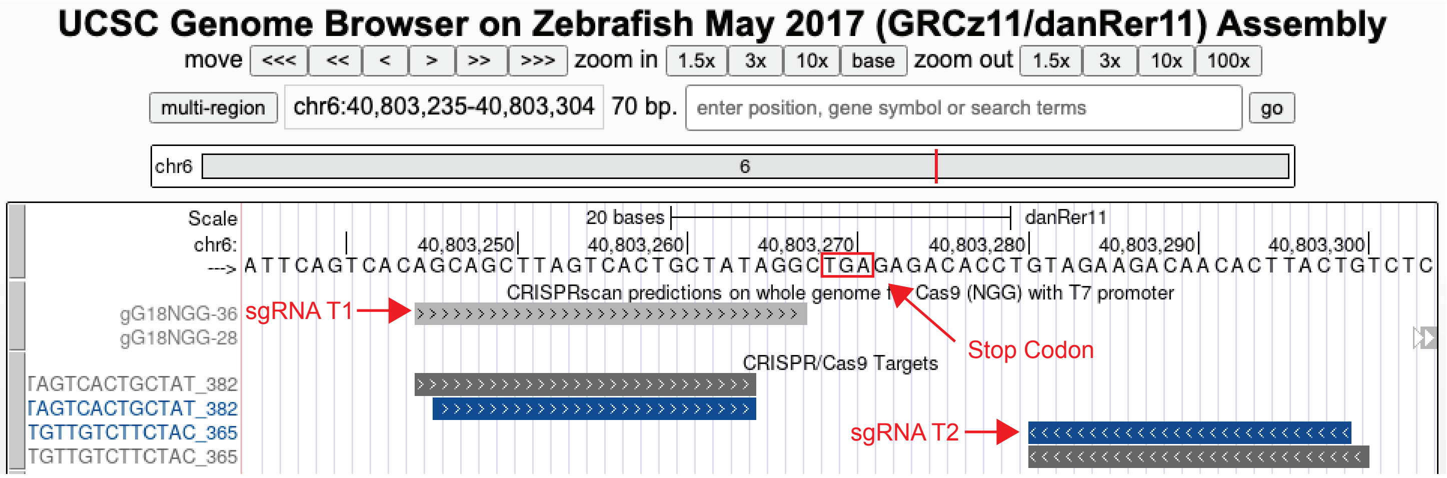

B

CRISPR-STAT analysis to determine activity level for sgRNA's

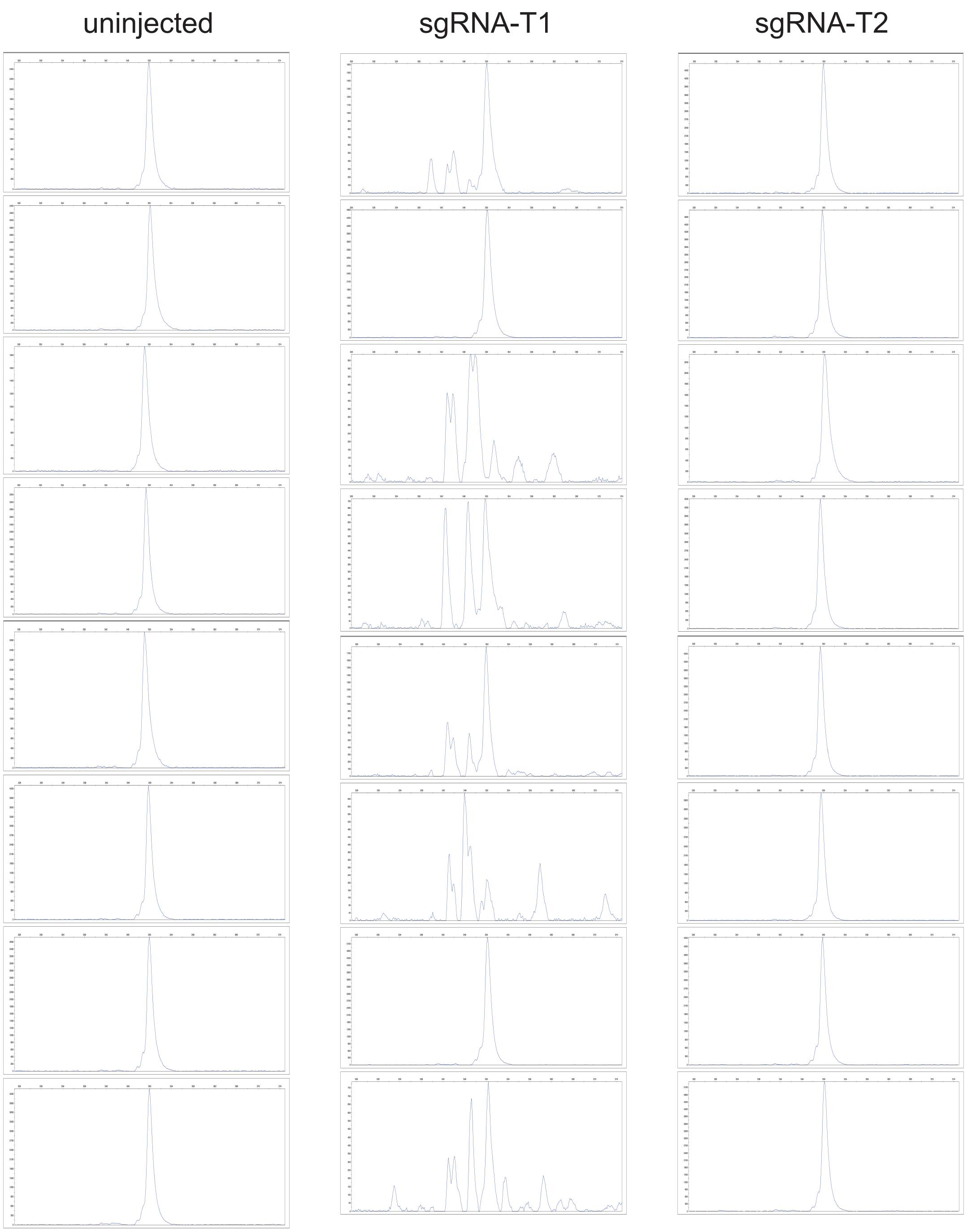

Figure S5: Design of *gata2b* ssODN and somatic and germline screening.

A. Detailed design of *gata2b* ssODN

```
ref seq   AATTACATCCTGCATTCAGTCACAGCAGCTTAGTCACTGCT------------------------------------ATAGGCTGAG
ssODN           ATCCTGCATTCAGTCACAGCAGCTTAGTCACTGCTATAGCCTACCCATACGACGTCCCAGACTACGCTTGAATAGGCTGAG
sgRNA                                    AGCAGCTTAGTCACTGCTATAGG


ref seq   AGACACCTGTAGAAGACAACACTTACTGTCTCCATTCTTTGGTTTTACTGTACAGGACATTTCATTAACATTTCAGATGTTTATTAT
ssODN     AGACACCTGTAGAAGACAACACTTACTGTCTCCATTCTTTGGTTTTACTGTACAGGACATTTCATTAACATTTCAGATGTT
sgRNA
```

B. Sequence of TOPO clone showing clean inseriton of desired knock-in sequence

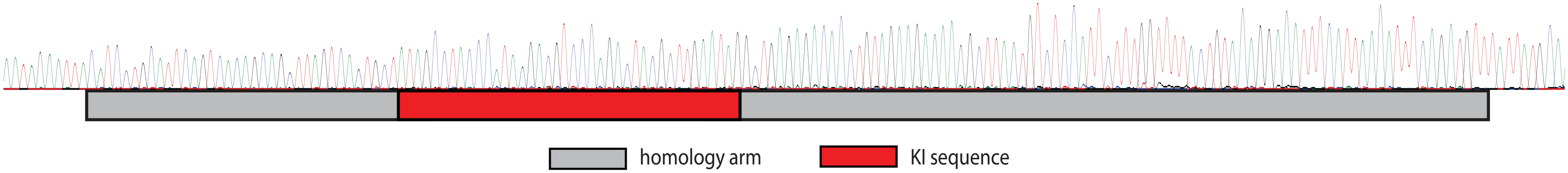

C. Representative plot from an F1 embryo heterozygous for the knock-in allele

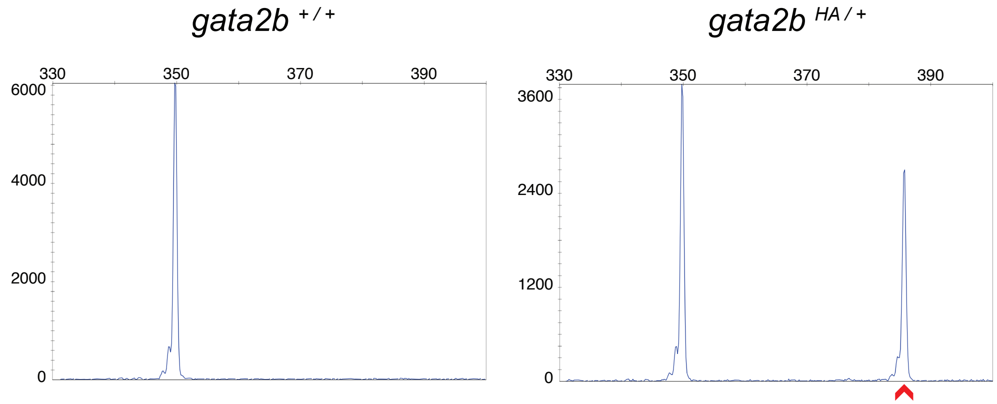

Figure S6: Sequence confirmation of *gata2b* $^{HA/HA}$ RT-PCR product.

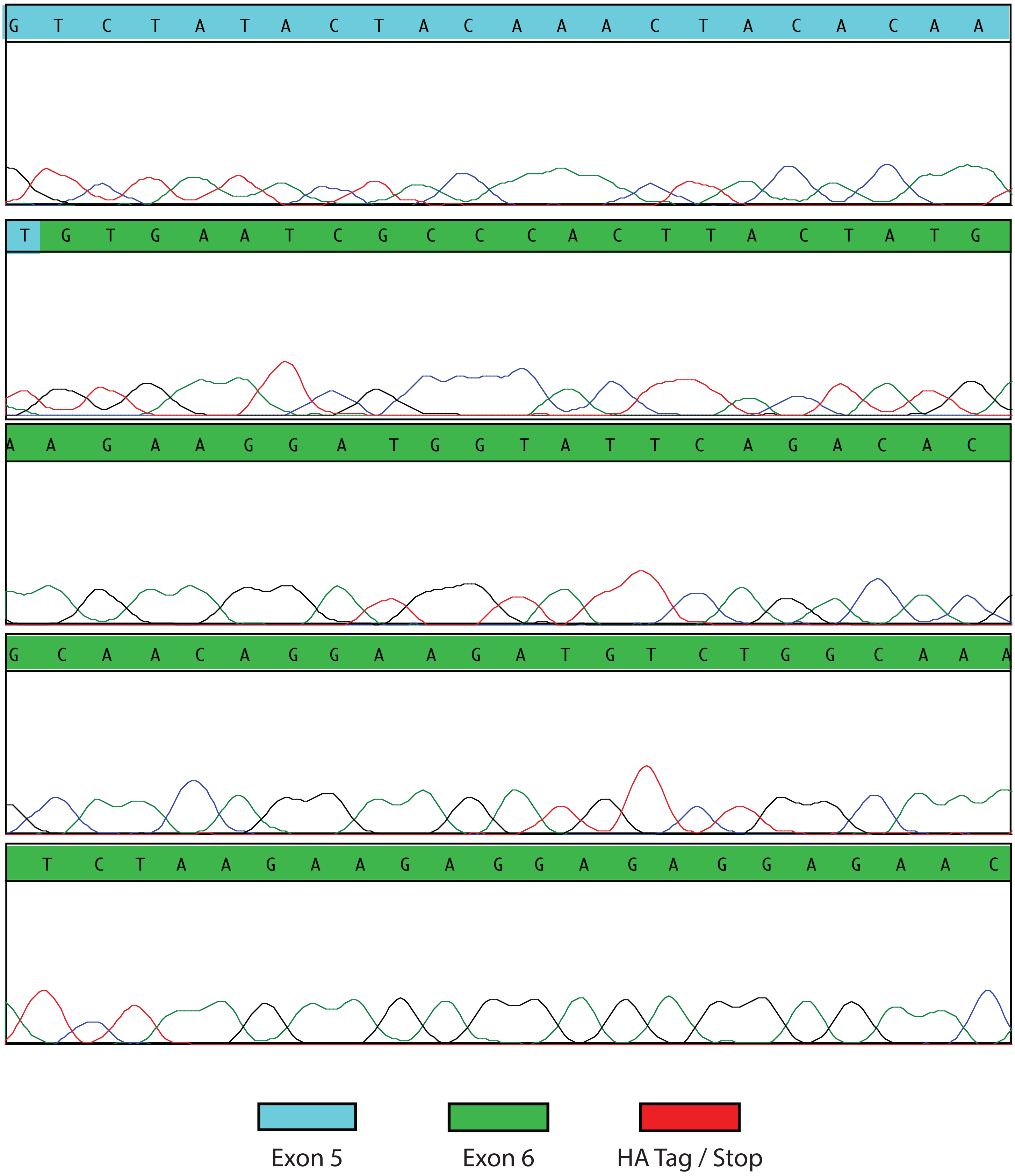

Figure S6 continued: Sequence confirmation of *gata2b* $^{HA/HA}$ RT-PCR product.

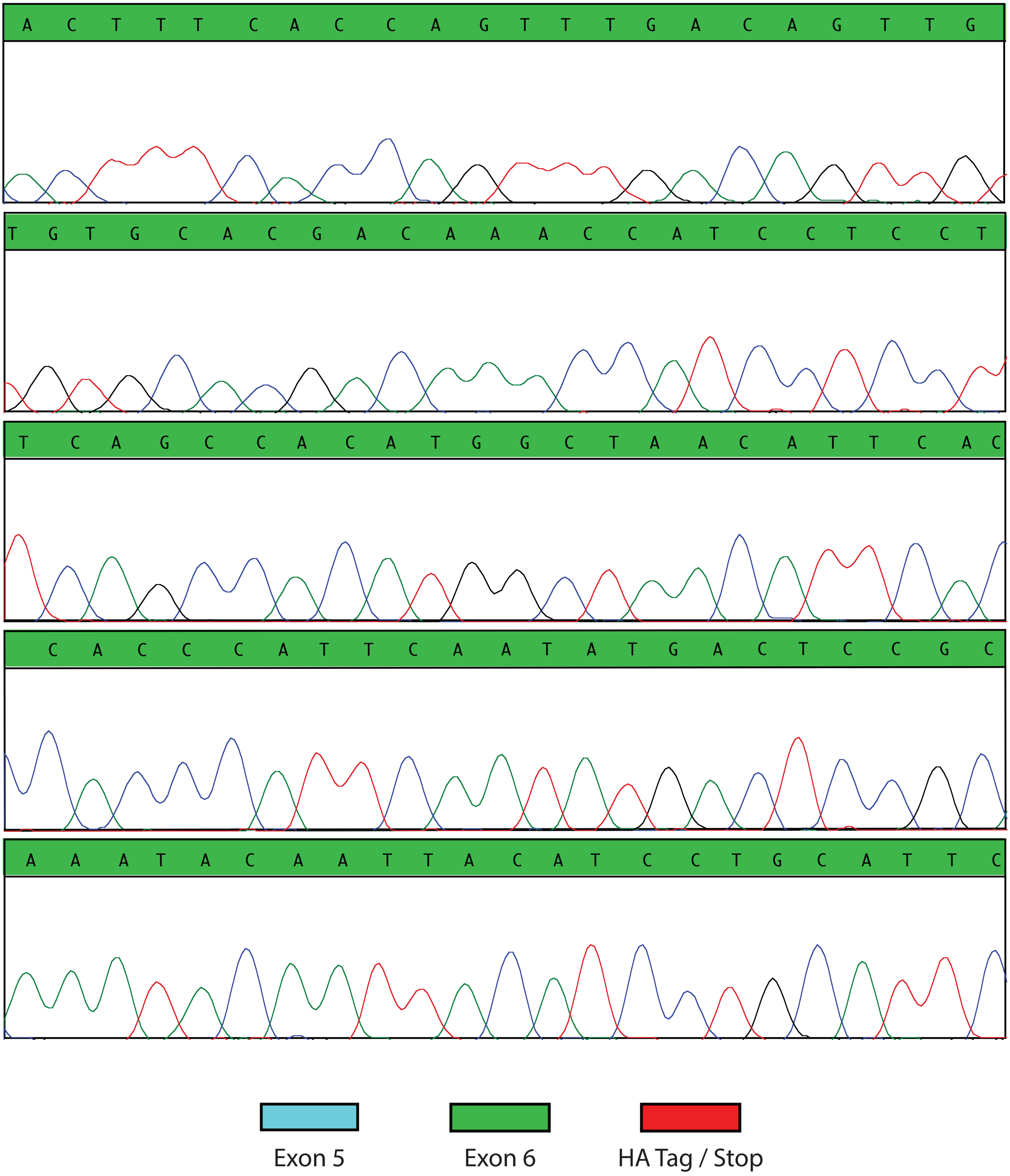

Figure S6 continued 2: Sequence confirmation of *gata2b* $^{HA/HA}$ RT-PCR product.

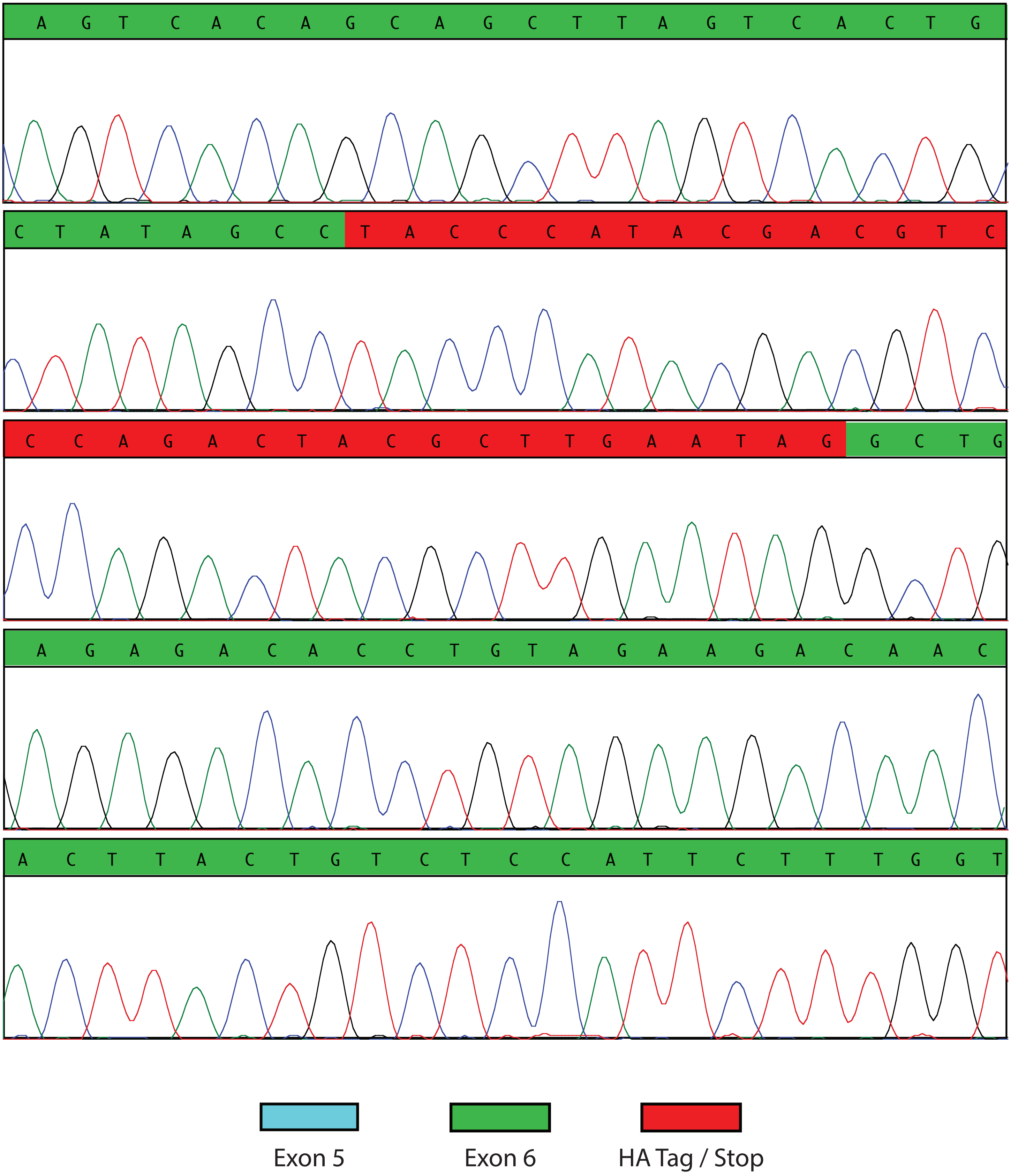

Figure S7: *gba* CRISPR selection and CRISPR-STAT analysis.

A

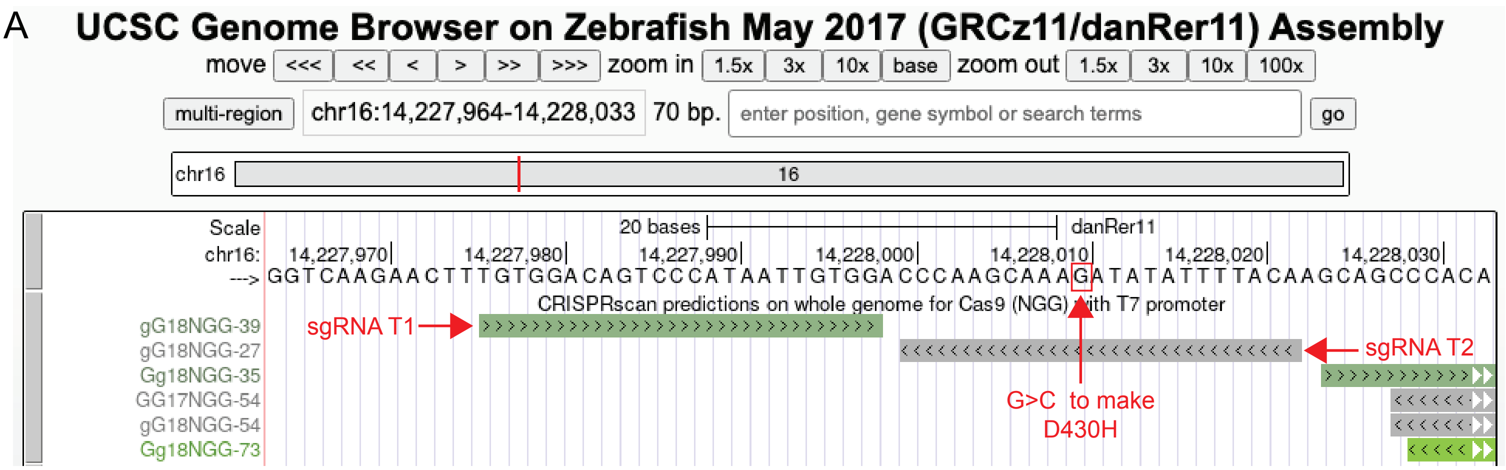

B

CRISPR-STAT analysis to determine activity level for sgRNA's

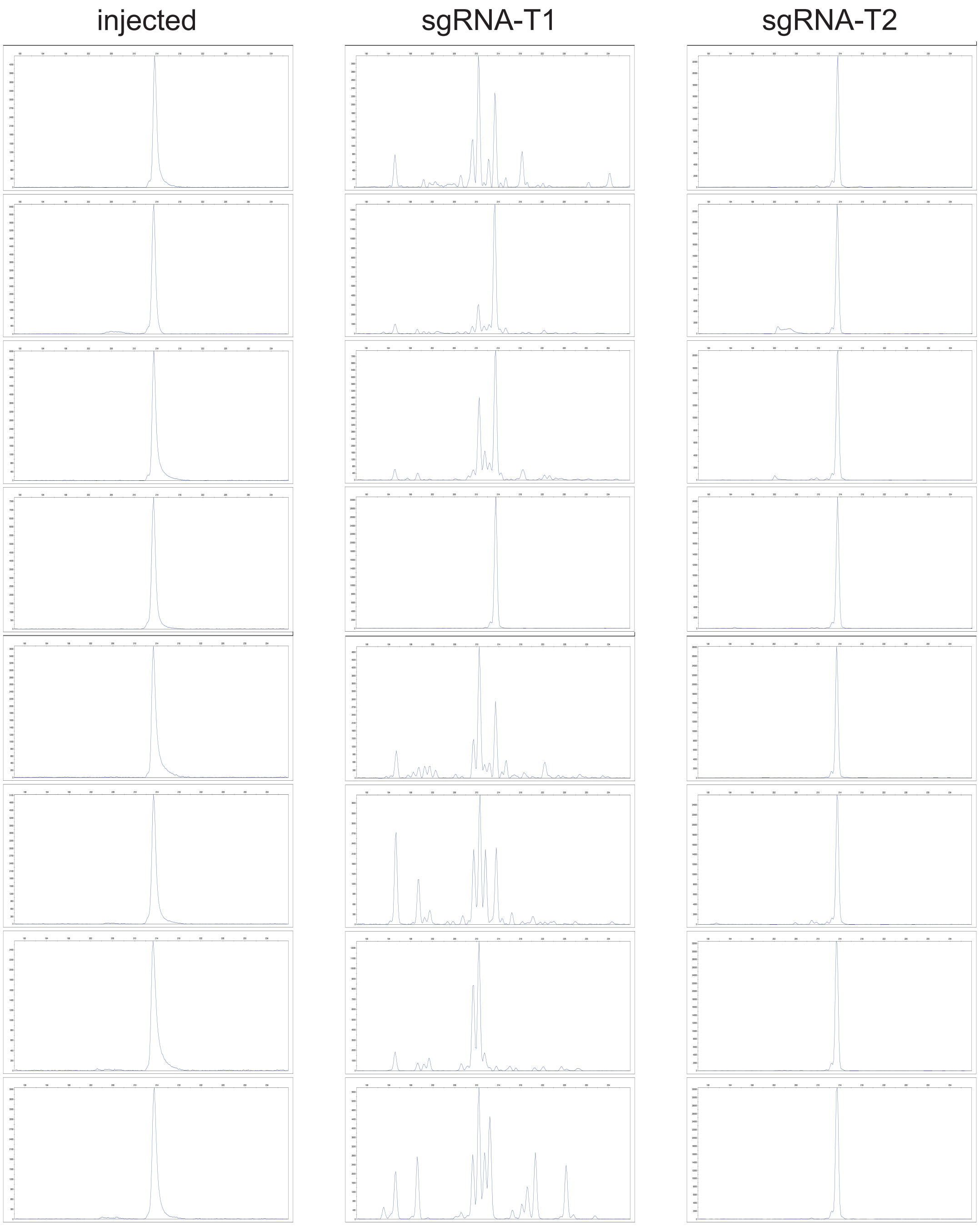

Figure S8: Design of *gba* ssODN and sequence confirmation of knock-in in injected embryos.

A. Detailed design of *gba* ssODN

```
ref seq         GAGGACCCAACTGGGTCAAGAACTTTGTGGACAGTCCCATAATTGTGGACCCAAGCAAAGATATATTTTA
our WT cohort   GAGGACCCAACTGGGTCAAGAACTTTGTGGACAGTCCCATAATTGTGGACCCAAGCAAGAATATATTTTA
ssODN                 CCAACTGGGTCAAGAACTTTGTGGACAGTCCCATAATTGTCGACCCAAGCAAACATATATTTTA
sgRNA                                    TGTGGACAGTCCCATAATTGTGG
sal1 restriction site                                       GTCGAC
```

```
ref seq         CAAGCAGCCCACATTCTACAGCATGGCCCACTTCAGGTTAGACTCGTTCAAAGCACCTTCAGTCCTGAAA
our WT cohort   CAAGCAGCCCACATTCTACAGCATGGCCCACTTCAGGTTAGACTCGTTCAAAGCACCTTCAGTCCTGAAA
ssODN           CAAGCAGCCCACATTCTACAGCATGGCCCACTTCAGGTTAGACTCGTTCAAAGCACCTTCAGTC
sgRNA
sal1 restriction site
```

B. Sequence of TOPO clone showing clean inseriton of desired knock-in sequence

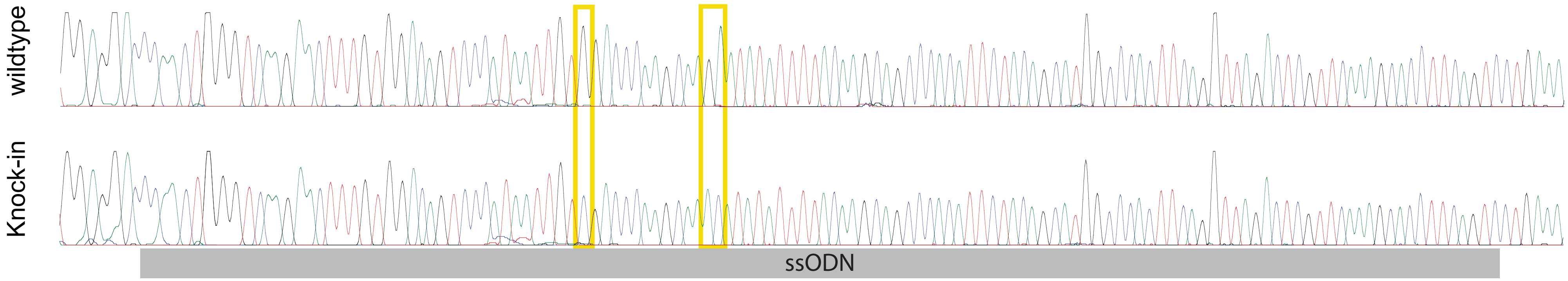

Supplement: Supplementary file 1 — Additional file 1: Table S1. Sequences of all primers and sgRNA's used in this study. Figure S1. tcnba CRISPR selection and CRISPR-STAT analysis. A Screenshot of the tcnba target region from UCSC genome browser using the CRISPRscan track showing possible sgRNAs near the stop codon. B Evaluation of individual embryos by CRISPR-STAT to determine activity levels. In all plots, the X-axis shows the size of the peaks and the Y-axis shows the peak height. sgRNA-T2 showed higher activity and was selected for knock-in experiments. Figure S2. Design of tcnba ssODN and somatic and germline screening. A Detailed design of the ssODN aligned with the ref sequence (chr5:30,618,013-30,618,151) and sgRNA used for generating the DSB. B Sequence chromatogram of a clone from an injected embryo positive for the knock-in peak confirming the insertion of the desired sequence as well clean and precise integration at each end of the ssODN. C Representative plot (X-axis showing the size of the peaks and the Y-axis showing the peak height) from an F1 embryo heterozygous for the knock-in allele (denoted by red arrowhead) compared to a WT embryo. Figure S3. Sequence confirmation of the tcnbaFLAG/FLAG RT-PCR product. Figure S4. gata2b CRISPR selection and CRISPR-STAT analysis. A Screenshot of UCSC genome browser using the CRISPRscan and ZebrafishGenomics tracks showing possible sgRNAs near the stop codon. B Evaluation of individual embryos by CRISPR-STAT to determine activity levels. In all plots, the X-axis shows the size of the peaks and the Y-axis shows the peak height. sgRNA-T1 showed higher activity and was selected for knock-in experiments. Figure S5. Design of gata2b ssODN and somatic and germline screening. A Detailed design of the ssODN aligned with the ref sequence (chr6:40,803,222-40,803,359) and sgRNA used for generating the DSB. B Sequence of a TOPO clone from an injected embryo positive for the knock-in peak confirming the insertion of the desired sequence as well clean and precis [file 12864_2022_8971_MOESM1_ESM.pdf]
