# Supplementary material for: A robust pipeline for efficient knock-in of point mutations and epitope tags in zebrafish using fluorescent PCR based screening
Source: BMC Genomics. 2022 Dec 7;23:810. doi: 10.1186/s12864-022-08971-1 (PMC9730659; doi:10.1186/s12864-022-08971-1)
Supplement: Supplementary file 2 — Additional file 2. Original images for gels shown in Figures 1D and 2D. [file 12864_2022_8971_MOESM2_ESM.pdf]

Figure 1- Supporting data: Expression of FLAG tag at *tcnba* locus by RT-PCR.

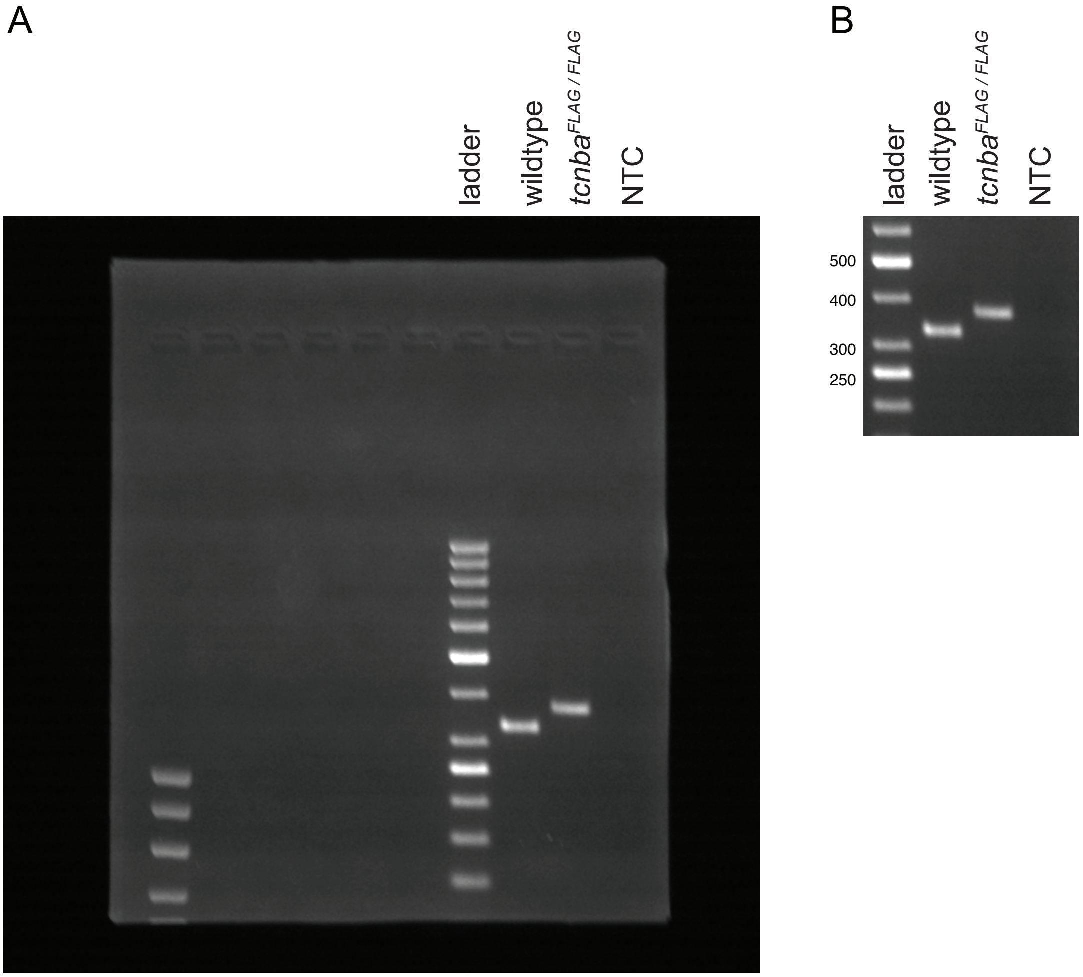

Figure 1 - Supporting data: Expression of FLAG tag at *tcnba* locus by RT-PCR. (A) Original gel image (B) cropped gel image as presented in figure 1D.

Figure 2- Supporting data: Expression of HA tag at *gata2b* locus by RT-PCR.

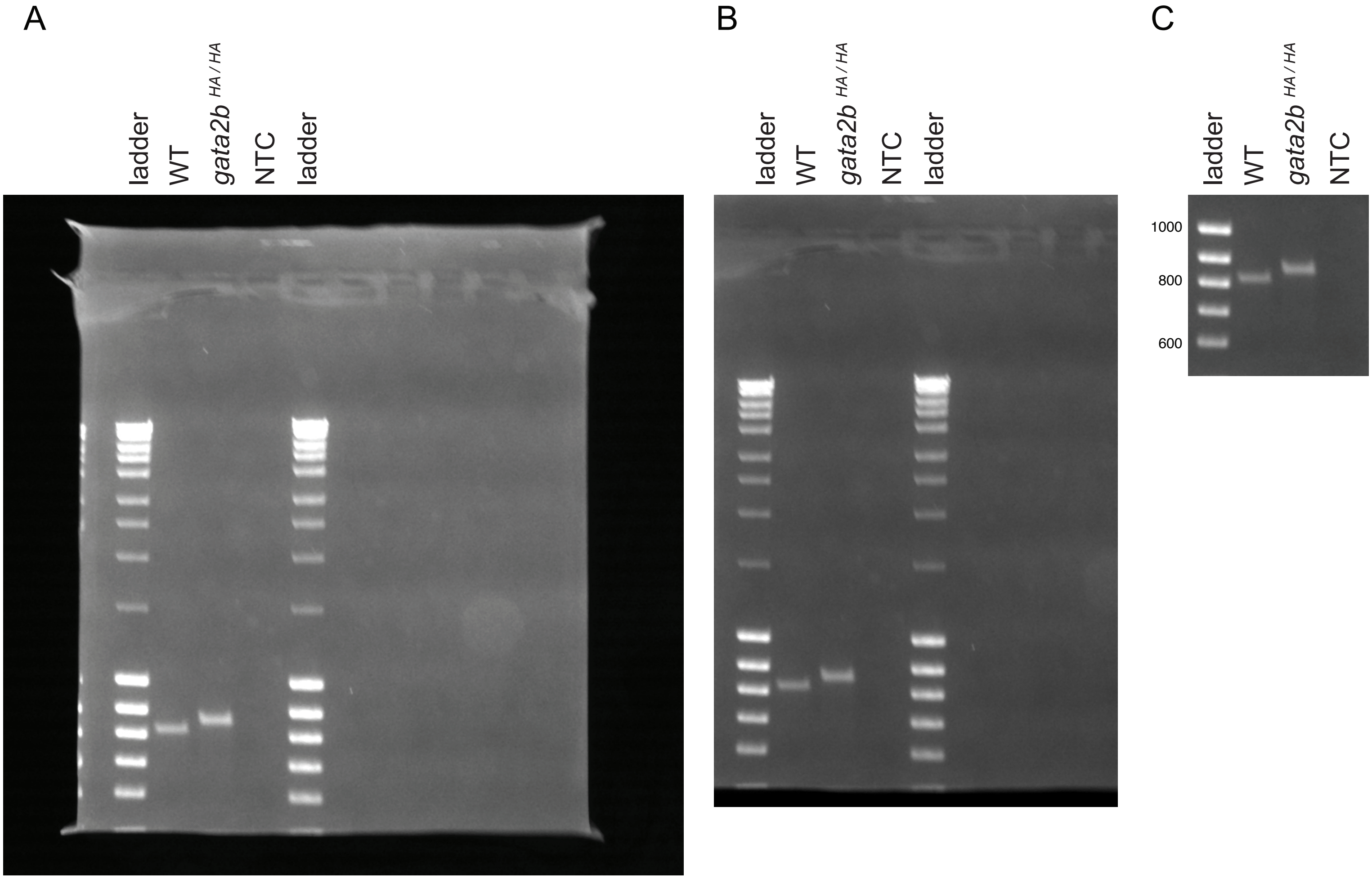

Figure 2 - Supporting data: Expression of HA tag at *gata2b* locus by RT-PCR. (A) Original gel image (B) Image processed with Azure Biosystems imaging system, version 1.6.4.1229, to crop and adjust brightness and contrast (C) Further cropping of processed image for presentation in figure 2D.
